# Metabolomic Analysis of the Effect of *Lippia origanoides* Essential Oil on the Inhibition of Quorum Sensing in *Chromobacterium violaceum*

**DOI:** 10.3390/antibiotics12050814

**Published:** 2023-04-26

**Authors:** Marlon Cáceres, William Hidalgo, Elena E. Stashenko, Rodrigo Torres, Claudia Ortiz

**Affiliations:** 1 Escuela de Medicina, Universidad Industrial de Santander, Bucaramanga 680002, Colombia; mayecaor@correo.uis.edu.co; 2 Escuela de Química, Universidad Industrial de Santander, Bucaramanga 680002, Colombia; 3 Escuela de Química, Centro de Cromatografía y Espectrometría de Masas (CROM-MASS), Universidad Industrial de Santander, Bucaramanga 680002, Colombia; elena@tucan.uis.edu.co; 4 Grupo de Investigación en Bioquímica y Microbiología, Universidad Industrial de Santander, Bucaramanga 680002, Colombia; rtorres@uis.edu.co; 5 Escuela de Microbiología y Bioanálisis, Universidad Industrial de Santander, Bucaramanga 680002, Colombia; ortizc@uis.edu.co

**Keywords:** bacterial communication, violacein, antibacterial compounds, microbial metabolomics

## Abstract

Bacteria can communicate through quorum sensing, allowing them to develop different survival or virulence traits that lead to increased bacterial resistance against conventional antibiotic therapy. Here, fifteen essential oils (EOs) were investigated for their antimicrobial and anti-quorum-sensing activities using *Chromobacterium violaceum* CV026 as a model. All EOs were isolated from plant material via hydrodistillation and analyzed using GC/MS. In vitro antimicrobial activity was determined using the microdilution technique. Subinhibitory concentrations were used to determine anti-quorum-sensing activity by inhibition of violacein production. Finally, a possible mechanism of action for most bioactive EOs was determined using a metabolomic approach. Among the EOs evaluated, the EO from *Lippia origanoides* exhibited antimicrobial and anti-quorum activities at 0.37 and 0.15 mg/mL, respectively. Based on the experimental results, the antibiofilm activity of EO can be attributed to the blockage of tryptophan metabolism in the metabolic pathway of violacein synthesis. The metabolomic analyses made it possible to see effects mainly at the levels of tryptophan metabolism, nucleotide biosynthesis, arginine metabolism and vitamin biosynthesis. This allows us to highlight the EO of *L. origanoides* as a promising candidate for further studies in the design of antimicrobial compounds against bacterial resistance.

## 1. Introduction

The prevalence of bacterial resistance to antimicrobial drugs has led researchers to discover new synthetic and/or natural antimicrobial compounds to treat various multi-resistant pathogens [1]. Factors that may contribute to bacterial resistance include the ability of bacteria to form biofilms and communicate through a mechanism known as quorum sensing [2,3]. Quorum sensing (QS) is a cell-density-dependent bacterial communication mechanism that is carried out through the secretion of signal molecules known as autoinducers [4,5]. When the concentration of signal molecules reaches a certain threshold, the expression of certain genes is promoted, causing differential phenotypic expression [6]. In addition, QS regulates a series of bacterial processes, such as biofilm formation, expression of virulence factors, motility, bioluminescence, spore formation, toxin production, and extracellular polysaccharide synthesis [7,8].

In general, *C. violaceum*, a Gram-negative bacterium, has been widely used in research related to QS due to its ability to produce violacein, a purple pigment encoded by the *vio* operon, whose expression is encoded by the QS mechanism [9]. Moreover, *C. violaceum* is considered an emerging human pathogen that has been associated with respiratory tract infections, liver abscesses, endocarditis, meningitis and fulminant sepsis [10,11].

Different antimicrobial approaches have been explored to avoid biofilm formation. Inhibition of QS has been identified as a possible antimicrobial target since it is associated with an increase in the pathogenicity of microorganisms [12]. Therefore, it is essential to search for new therapeutic candidates that interfere with the QS mechanism and thus counteract bacterial pathogenicity. Plant-derived candidates stand out as the most-studied natural QS inhibitors. Plant extracts have been shown to inhibit QS mainly due to their structural similarity to some signals related to the QS mechanism, such as AHL; the ability of plant compounds to degrade signals from LuxR/LuxS receptors has also been reported [13]. Among these, EOs are promising antimicrobial natural products. They are volatile natural mixtures of compounds consisting mainly of terpenoid and phenolic compounds, distilled from different parts of the plant and exhibiting both antimicrobial and anti-QS activities [14,15,16]. In our previous study, a thymol–carvacrol chemotype *Lippia origanoides* EO (LOT-II) was a potent QS inhibitor against *C. violaceum* [17]. Nevertheless, the influence of LOT-II on the metabolic fingerprint and the potential inhibition mechanism remains unclear. Additionally, metabolomics research has been widely used to unveil metabolic changes in cells under certain physiological conditions. Thus, our study aimed to explore the anti-QS activity of fifteen EOs on *C. violaceum* CV026 and, through a metabolomics approach, elucidate the plausible mechanisms of action of the EO with the highest biological activity.

## 2. Results

### 2.1. Essential Oil Distillation and Chemical Characterization

The EOs distilled from the 15 fresh plants under study were a complex mixture of monoterpenoids, sesquiterpenoids and phenolic compounds. The EO distillation techniques used were microwave-assisted hydrodistillation (MWHD) or steam distillation (SD), depending on the amount of plant material available. The EO yields obtained appear in Table 1. The EO yields (0.1–0.8%, *w*/*w*) obtained via MWHD from *Steiractinia aspera* (0.1%), *Elaphandra quinquenervis* (0.2%), *Hyptis dilata* (0.8%), *Lippia micromera* (0.6%), *Piper reticulatum* (0.1%), *Ageratina popayanensis* (0.3%), and *Ocimum campechianum* (0.4%) did not exceed 1%, as well as the yields achieved via steam distillation from *Turnera diffusa* (0.3%), *Calycolpus moritzianus* (0.2%), *P. aduncum* (0.4%), *Satureja viminea* (0.6%), *Psidium santorianum* (0.5%), *Varronia curassavica* (0.2%), *O. basilicum* (0.2%), and *L. origanoides* (0.7%). The highest EO yields were reached by distilling *Hyptis dilata* (Lamiaceae) and *L. origanoides* (Verbenaceae) plants, both native to tropical America and abundant in Colombia.

The chemical compositions of the EOs were established using the GC/MS method and were based on both chromatographic data (t_R_, linear retention indices, LRIs) and mass spectral experimental data (molecular ions, isotope abundance ratios, fragmentation patterns) compared with those from MS databases and the literature. The chemical identity of the main EO components was confirmed by comparison of their t_R_, LRIs (Table S1, Supplementary Materials) and mass spectra with those of the standard compounds analyzed under the same GC/MS operation conditions. The main volatile secondary metabolites distilled from the 15 plants studied appear in Table 1, together with their relative amounts (%), botanical information (voucher numbers), and EO distillation methods and yields (%, *w*/*w*). Various volatile metabolites appeared “repetitively” in the different EOs studied here, including α- and β-pinenes, limonene, 1,8-cineole, *p*-cymene, germacrene D, and *trans*-β-caryophyllene and its oxide (Table S1). The *Ocimum* spp. were rich in phenylpropanoids, such as estragole *(O. basilicum)* and eugenol *(O. campechianum),* while the *Lippia* spp. studied possessed predominantly phenolic monoterpenes, i.e., thymol and carvacrol and their methyl ethers. *Steiractinia aspera*, *Turnera diffusa*, *Elaphandra quinquenervis*, and *Varronia curassavica* contained monoterpene and sesquiterpene hydrocarbons. In the Supplementary Materials, the EO chromatographic profiles of the 15 EOs studied with the chemical structures of their main volatile secondary metabolites are reported, as well as photos of some of the plants (Figures S1 and S2, Supplementary Materials).

### 2.2. Antibacterial Activity of EOs

The antimicrobial activities of the EOs against CV026 were determined using the broth microdilution method [13]. The results of both the minimal inhibitory concentration of each EO (MIC_50_) and the minimal bactericidal concentration (MBC) are summarized in Table 2. Six of the fifteen EOs evaluated presented antimicrobial activity below 1 mg/mL. Among them, the EO from *L. origanoides* showed the highest bactericidal activity (0.5 mg/mL). In contrast, EOs distilled from *Steiractinia aspera* and *Elephandra qinquenervis* did not show significant results.

### 2.3. Anti-Quorum-Sensing Activity

**Figure 1:**
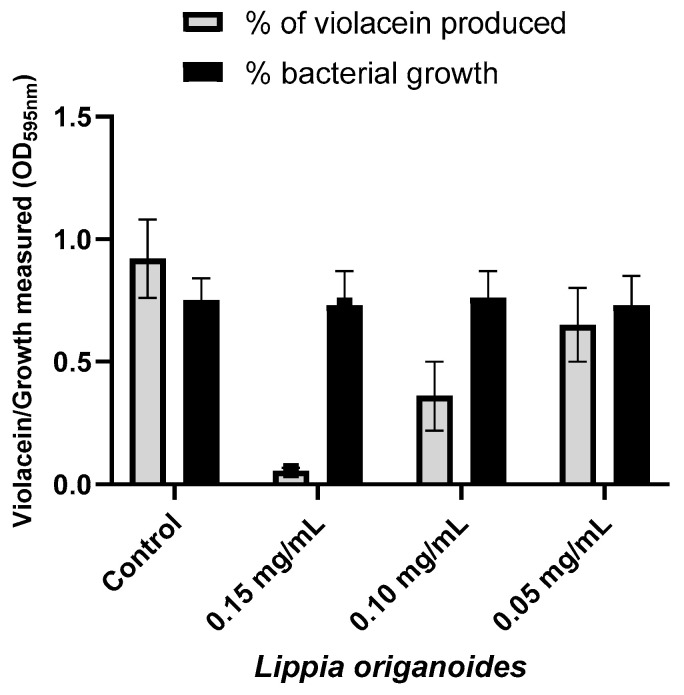
Violacein production in the CVO26 bacterial strain during treatment with *L. origanoides* EO. Data are presented as the mean ± SD of absorbance (at 595 nm).

The MIC_50_ values of the EOs obtained for the CVO26 bacterial strain ranged from 0.37 to 3.0 mg/mL (Table 2). A series of sub-MIC_50_ concentrations were selected for tracking violacein production in the CV026 strain. A significant reduction of 94.0% in pigment production (violacein) was observed when CV026 was treated with *L. origanoides* EO (Figure 1). To a lesser extent, EOs distilled from *Ocimum basilicum* and *Varronia curassavica* exhibited reductions of 85% and 72%, respectively. The results of anti-QS activity from all EOs assessed are listed in the Supplementary Materials (Table S2). Additionally, bacterial viability was evaluated by counting colony-forming units (CFU/mL) in the culture plate to test whether there was an effect on bacterial growth. The results did not show significant differences in terms of bacterial growth between the control and treated samples (Table S3, Supplementary Materials).

Subsequently, the essential oil with the highest antimicrobial and anti-quorum-sensing activity was selected; in this case, the essential oil was the one extracted from the *Lippia origanoides* plant, with which the metabolomic analysis was continued to determine its possible mechanism of action.

### 2.4. Metabolomics Analysis

**Figure 2:**
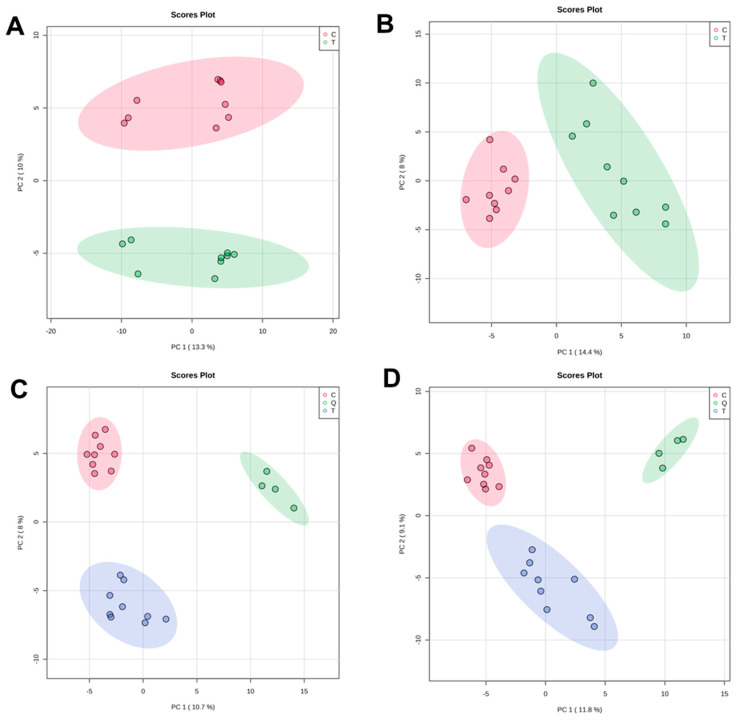
PCA of features obtained from LC/MS of CV026 with *L. origanoides* EO treatment. (**A**) PCA for LC/MS operated in positive ion mode. (**B**) PCA for LC/MS operated in negative mode. (**C**) PCA including QC samples for LC/MS operated in positive mode. (**D**) PCA including QC samples for LC/MS operated in negative mode.

The data set for LC/MS analysis carried out in the positive and negative acquisition modes had 4188 and 2355 features, respectively. After feature extraction analysis, feature inspection and filtering, the sizes of these data sets were reduced to 2289 and 1518, respectively. To evaluate the performance of the analytical platform used, principal component analysis (PCA) was carried out (Figure 2A,B). The results had good grouping of quality control (QC) samples (Figure 2C,D), which indicates high-quality data acquisition reproducibility; therefore, the separation between the groups is related to biological variations. PCA models were constructed, with features corresponding to coefficients of variation less than 30%.

**Figure 3:**
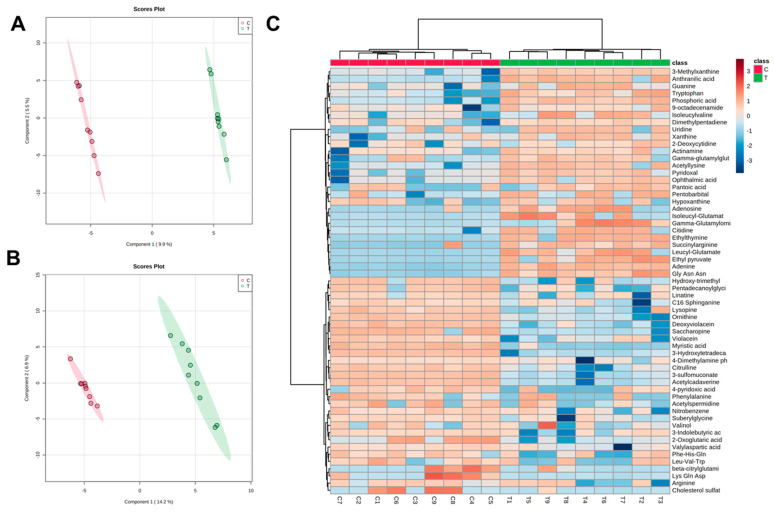
PLS-DA and heatmap analysis of different metabolites. (**A**) PLS-DA analysis of control and treated cells. Each dot represents the biological replicate analysis of a sample in positive mode. (**B**) PLS-DA plot in negative mode. (**C**) A heatmap was constructed based on the differential metabolites in the treated group (T) and control group (C) (*n* = 9). The color key indicates metabolite abundance (orange: up-regulation; blue: down-regulation). Rows: metabolites; columns: samples. The control and treatment group are colored red and green, respectively.

Supervised PLS-DA was applied for modeling differences between the groups, which showed clear separation of control and treated samples (Figure 3A,B) with high quality, evidenced by good values of explained variance (R^2^) and predictive variance (Q^2^). Subsequently, the PLS-DA models were validated by using cross-validation to estimate the predicted data. According to uni- and multivariate statistical analyses, variable importance in projection (VIP), fold change (Log2FC) and statistic *p*-values, a total of 55 putatively significant metabolites (under both ionization modes) were identified via the comparison of their mass spectra and retention times with those reported in databases and the literature. The results of the identified statistically significant metabolites are summarized in Table 3.

The main statistically significant metabolites found in *C. violaceum* after EO treatment were classified according to their role in the biosynthetic pathways of bacterial metabolism (Table 4). These metabolites belonged to tryptophan metabolism (4 metabolites detected), nucleotide biosynthesis (10 metabolites detected), arginine metabolism (5 metabolites detected), vitamin biosynthesis (3 metabolites detected), lysine metabolism (3 metabolites detected), peptides (9 metabolites detected), organic acids (4 metabolites detected), lipids (2 metabolites detected), phenylalanine metabolism (2 metabolites detected) and others (13 metabolites detected) according to metabolic pathway analysis. Among the associated metabolic pathways, the increase in metabolites related to the metabolism of nucleotides and those with precursors in the formation of violacein in the sample treated with EO is relevant. On the other hand, a decrease in the metabolites associated with arginine metabolism and the final products of tryptophan metabolism was evidenced in the treated sample. The heatmap of differential metabolites showed that the abundance of *C. violaceum* metabolites changed significantly after EO administration, with 28 metabolites up-regulated and 27 metabolites down-regulated (Figure 3C).

## 3. Discussion

Among the EOs analyzed, the highest antimicrobial activity against CV026 was obtained with the LOT-II and OB EOs, with an MIC_50_ of 0.37 mg/mL. However, LOT-II EO at a concentration of 0.5 mg/mL was able to completely inhibit the bacterial population. These results show that the MIC_50_ values for the LOT-II and OB EOs were lower than those reported in the literature for other EOs. For instance, Noumi et al. (2018) determined a MIC_50_ of 10 mg/mL for EOs from *Melaleuca alternifolia* [18]. In other investigations [19,20], EOs from *Carum copticum* and *Mentha suaveolens* were evaluated and had MIC_50_ values of 0.6 mg/mL and 1.5 mg/mL, respectively, against CV026.

Biological tests for evaluating the inhibition of violacein production were performed using the biosensor bacterium CV026, a wild-type mini-T5 mutant that lacks the AHL synthase enzyme encoded by the *cvil* gene and therefore can only produce violacein in response to externally supplied AHL signal molecules [21]. Although violacein production has been widely studied as a biological trait that is regulated by QS, other phenotypes such as elastase and cyanide production are also controlled through QS communication, mediated by the AHL signal [22,23,24].

Inhibition assays in the production of violacein caused by the EOs against the CV026 strain were performed by using sub-inhibitory concentrations to validate that the mechanism of action of EO is mediated by the blockage of QS instead of decrease in the microbial growth of planktonic cells (Figure 1). Violacein production in CV026 was dramatically affected when different sub-inhibitory concentrations of the LOT-II, OB and VC EOs were assessed. Among these three EOs, LOT-II EO exhibited the highest inhibition, with a decrease of 94.0% at a concentration of 0.15 mg/mL. Thymol and carvacrol correspond to the major constituents from LOT-II EO and, therefore, biological activity could be attributed to these compounds as has been previously reported, with reductions in violacein production of 8.35% and 12.11%, respectively [25]. However, such differences in the anti-QS activity of thymol and carvacrol against the CV026 strain contrast with our findings, suggesting that the observed effect does not depend only on the major components of the LOT-II EO.

A possible mechanism of action of the EO with the highest inhibitory activity of quorum sensing was proposed based on the analysis of the main metabolic pathways affected in CV026 by the effect of the LOT-II EO, using information obtained from metabolites detected with statistically significant differences [26]. Therefore, the detected metabolites were associated with their respective metabolic pathways and the analysis of the possible metabolic effects produced by LOT-II EO is shown in Table 4. Here, the effect caused by LOT-II EO on the synthesis of violacein (with the highest VIP value, Table 3) is of special interest due to its direct correlation with bacterial communication through the QS mechanism in CV026 [27]. The CV026 QS-system consists of two components called *CviI* and *CviR*, which respond to high-affinity acyl-homoserine lactone (AHL) molecules [28,29]. With increasing bacterial cell density, AHL molecules bind to cognate receptors, forming a complex that regulates the expression of target genes (including the ABCDE operon) and induces the formation of the violacein pigment [30]. It is important to note that violacein synthesis, as a product of the QS mechanism in CV026, is associated with the regulation of virulence factors such as Type II metalloproteases (TIISS) and Type III secretion systems (TIIISS), swarm motility, and the synthesis of exopolysaccharides, flagellar proteins, collagenase, chitinase and cytolytic toxins [31,32,33]. Therefore, blocking violacein production in the bacterial population without affecting cell viability can be a feasible alternative to treat bacterial resistance, highlighting our findings with EO from LOT-II as a promising candidate for future therapeutic agent development.

**Figure 4:**
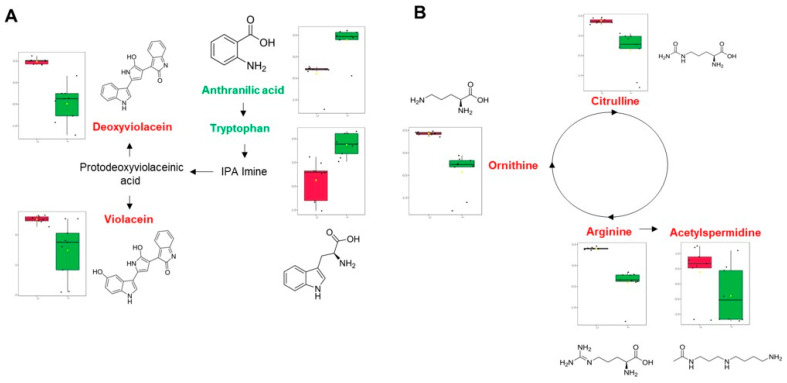
Visualization of the main metabolic pathways affected after treatment with LOT-II EO on CV026. (**A**) Tryptophan metabolism. (**B**) Arginine metabolism. Colored metabolites indicate relative abundance (green: up-regulation; red: down-regulation). Red and green boxes correspond to control and treated samples.

Analysis of violacein biosynthesis showed a clear effect on the biocatalytic process carried out by violacein synthase, which catalyzes hydroxylation at position 16 of protoviolacein and deoxyprotoviolacein to produce violacein and deoxyviolacein, respectively, when CV026 is treated with LOT-II EO [34,35]. However, tryptophan, a precursor in the synthesis of violacein, was increased in the samples treated with the EO, which suggests truncated metabolism in the cell of not only tryptophan, but also its precursor, anthranilic acid (Figure 4A). In addition, this suggests that bacterial cells could redirect their metabolisms to other metabolic pathways to maintain cell viability [36]. This is relevant because even though QS was inhibited, the microorganism was still viable, which indicates that the vital functions of CV026 did not present major effects at the metabolic level.

In addition to the inhibition of violacein production, there was also evidence of a decrease in metabolite concentrations associated with arginine metabolism, including metabolites related to the urea cycle and polyamine synthesis (Figure 3B). In this case, there was an effect on the production of the polyamine acetylspermidine, a compound related to the maintenance of cellular homeostasis conditions, such as transcription [37], translation [38], biofilm formation [39], and resistance to oxidative stress [40]. On the other hand, the effect on the expression of proteins associated with the metabolism of arginine has been previously associated with a reduction in violacein biosynthesis [41]. To our knowledge, this is the first report about the effect of an EO on the inhibition of quorum sensing in *C. violaceum* through a metabolomic approach. Here, we could evidence an effect on violacein and arginine metabolism (Figure 3), with a consequent decrease in the expressions of citrulline, ornithine, acetylperimidine and arginine, which affects the homeostasis of nitrogenous compounds without affecting cell viability.

**Figure 5:**
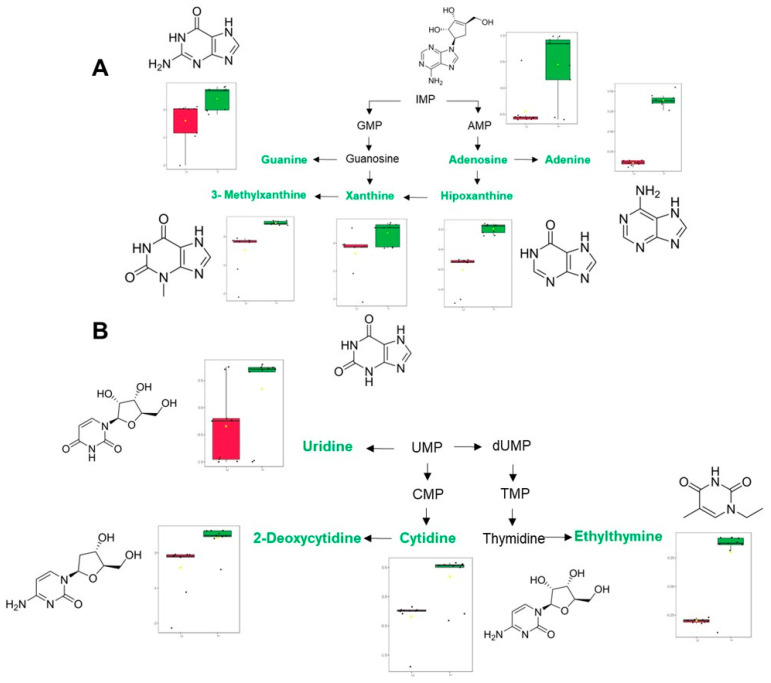
Visualization of the main metabolic pathways associated with nucleotides and their changes after treatment with EO. (**A**) Purine metabolism. (**B**) Pyrimidine metabolism. Colored metabolites indicate relative abundance (green: up-regulation; red: down-regulation). Red and green boxes represent control and treated samples.

Nucleotide metabolism was also affected by the treatment, especially that associated with metabolites related to purine and pyrimidine catabolism, which were found to be increased by the effect of LOT-II EO (Figure 5A,B). In this case, purine metabolism plays a pivotal role in biofilm formation, which has been evidenced in different studies on various bacterial models, suggesting that nucleotide biosynthesis is essential for proper biofilm formation [42,43,44,45]. Moreover, changes in the expression of signaling molecules derived from nucleotides (such as cAMP and c-di-GMP) could also be caused [46,47]. Finally, EO from LOT-II affected the metabolism of pyrimidines at the nucleic acid level in CV026. This may have some correlation with dysregulations in virulence related to QS in the microorganism, because pyrimidine metabolism has been associated with QS regulation in similar bacterial models [48]. Because metabolic studies related to CV026 are scarce, association, analysis and comparison of the obtained results are limited. Therefore, in further studies, we will carry out specific analyses focused on certain metabolites of CV026 to better understand the variations in the metabolism of CV026 produced by LOT-II EO.

## 4. Materials and Methods

The plants used in this study were harvested from experimental plots located in the Agroindustrial Pilot Complex of CENIVAM (National Center for Research in Agroindustrialization of Tropical Medicinal Aromatic Plants) at the Industrial University of Santander (Bucaramanga, Colombia). *C. violaceum* (CV026) was kindly donated by Dra. Nohora Rueda from the University of Santander—UDES, Bucaramanga, Colombia.

### 4.1. Plant Material

The plant material used in this study was gathered during botanical outings carried out in 2019–2020 at different sites of Santander (near Bucaramanga, the municipalities of Villa Nueva, Jordan, Zapatoca, and Betulia, or at San Vicente de Chucurrí, Santander State, Colombia) under an official government permit to access genetic resources and derivatives of bioprospecting (N° 270) given by the Ministry of Environment and Sustainable Development of Colombia. Taxonomic identification of the plants under study was carried out in the National Herbarium at the National University of Colombia (COL, UN, Bogotá) as well as, and predominantly, in the Herbarium of the Industrial University of Santander (UIS, Bucaramanga), directed by Associate Professor Andrés Felipe Castaño González, botanist, PhD. The plant exsiccatae and their voucher numbers (Table 1) were deposited in each herbarium. The gathered plants were propagated in the experimental plots (Figure S1, Supplementary Materials) of the Agroindustrial Pilot Complex and Botanical Garden *(“Un mundo en un jardín”* in Spanish, “A world in a garden”) at CENIVAM (National Centre for Research in Agroindustrialization of Tropical Medicinal Aromatic Plants at the Industrial University of Santander, Bucaramanga, Colombia). The plant material was propagated via cuttings or seeds according to the plant species. Plant cultivation (ex situ collection) was maintained under controlled agricultural conditions (temperature 26–28 °C, relative humidity 75–80%) respecting the principles of good agricultural practice. Only healthy and undamaged plant material was used for distillation. The plants were collected mostly before their flowering. The fresh plant material, previously chopped, was used immediately for distillation.

### 4.2. Essential Oil Distillation

According to the plant material available and its texture (i.e., herbs, stems, shrubs), the distillation process occurred by two methods, as follows: (1) microwave-assisted hydrodistillation (MWHD) and (2) steam distillation (SD). MWHD has been described extensively in our previous works [49,50]. Briefly, for MWHD, ca. two hundred grams of plant material and 200 mL of water were used, placed in a 2 L round bottom flask ubicated in a conventional microwave oven (Samsung MG32J5133AG, 1200 W, 2.45 GHz) and coupled to a classical Clevenger apparatus with a Dean–Stark reservoir. The MW oven was used at 60% power, for one hour, in 15 min intervals (15 min × 4). The essential oil obtained was separated by decantation, filtered, and dried using anhydrous sodium sulfate (Na_2_SO_4_).

The steam distillation occurred in a 0.4 m^3^ stainless-steel alembic, designed in our research center, CENIVAM, using 80 kg of plant material and a shell and tube condenser. The alembic was connected to an industrial boiler (6 BHP, *Tecnik*, Bogotá, Colombia) and distillation was carried out at 80 psi (*ca.* 550 kPa) vapor pressure for 1.5–2 h according to the type of plant material used. The vapor flow was 0.8 L/min. After its condensation, the EO was separated from a hydrosol in a stainless-steel Florentine flask (40 L), filtered and dried using anhydrous Na_2_SO_4_. For the gas chromatographic analyses (GC/MS), each EO was diluted (25%) in dichloromethane (CH_2_Cl_2_), and the solution was analyzed using GC/MS immediately after its preparation.

### 4.3. Essential Oil GC/MS Analyses

A description of the EO analysis via GC/MS appeared in our previous works [51,52]. Briefly, two capillary GC columns of the same dimensions (60 m longitude, 0.25 mm internal diameter, 0.25 µm film thickness) but different polarities (nonpolar, DB-5MS, with a 5% Ph-PDMS and polar, DB-WAX, with a PEG, stationary phases) were used. Both capillary columns were purchased from J&W Scientific (Folsom, CA, USA.). An Agilent Technologies (AT) gas chromatograph GC 6890 Plus (AT, Palo Alto, CA, USA.) coupled to a mass selective detector MSD 5973 Network (AT, Palo Alto, CA, USA.) was employed for the EO GC/MS analysis. An ionization chamber was held at 250 °C and 3.5 × 10^−6^ Torr vacuum pressure. Electron ionization (EI, 70 eV) was employed. For the ion separations and measurements, a single quadrupole mass filter was used at 150 °C, the mass range was set at *m*/*z* 40–400, and the acquisition rate was 3.58 scans/s. The performance of the GC/MS system was checked each second day via an autotune program using a perfluorotributylamine (PFTBA) analytical standard for the tuning and calibration of the mass axes. The electromultiplier was set at 1560 V according to the value optimized by the calibration procedure (autotune).

Helium (He) was used as a carrier gas (99.995%, gas AP, Messer, Bogotá, Colombia), and the initial He pressure in the column head was 113.5 kPa. During the chromatographic analysis, the He head pressure was automatically changed according to the column temperature to maintain a constant column flow at 1 mL/min. The samples were injected at 250 °C, using an automatic injection system, in a split mode (30:1). The transfer line temperature was set at 300 °C for the nonpolar (DB-5MS) column and 230 °C for the polar (DB-WAX) column. The GC oven temperature programming was as follows: from 45 °C (5 min) to 150 °C (2 min) at 4 °C/min, then to 300 °C (10 min) at 5 °C/min for the nonpolar DB-5MS column, and from 50 °C (5 min) to 150 °C (7 min) at 4 °C/min, then to 230 °C (50 min) at 4 °C/min for the polar DB-WAX column. All experimental data (GC peak integration, mass spectra, library search) were processed using MSD ChemStation G1701DA software (AT, Palo Alto, CA, USA).

Compound presumptive identification was carried out using chromatographic criteria (t_R_, linear retention indices, LRI) and mass spectrometric criteria (molecular ions, isotopic ratios, fragmentation patterns). A comparison of the experimental mass spectra was performed with those from the available databases (Wiley 2008, NIST 2017 and Adams 2007). Peak matching between the sample and the database mass spectra greater than 95% was accepted for positive compound identification. The LRIs were experimentally measured on both nonpolar and polar columns, using the calculating formula and criteria described thoroughly in the literature [53,54,55]. For LRI calculation, each EO sample was coinjected with the standard *n*-paraffin mixture C_5_–C_25_ purchased from Sigma-Aldrich (St. Louis, MO, USA). For EO component confirmatory identification, the standard compounds (α-pinene, β-pinene, camphene, sabinene, β-myrcene, α-phellandrene, *p*-cymene, limonene, 1,8-cineole, g-terpinene, linalool, α-thujone, isopulegol, α-terpineol, thymol, carvacrol, eugenol, *trans*-β-caryophyllene, α-humulene, germacrene D, caryophyllene oxide, β-eudesmol, guaiol, benzyl benzoate, 98–99% purity) were purchased from Sigma-Aldrich (St. Louis, MO, USA).

### 4.4. Determination of Antimicrobial Activity

The in vitro antimicrobial activity of the EOs was carried out as previously described [17]. Briefly, the minimal inhibition concentrations (MICs) of the EOs were determined using the broth microdilution method for bacteria in a 96-well microplate. The EOs were dissolved in dimethyl sulfoxide (DMSO) for molecular biology. Serial dilutions of the EOs were prepared ranging from 0.15 up to 3.0 mg/mL, for a final volume of 100 µL per well. All experiments were conducted with a maximum of 1% (*v*/*v*) DMSO in solution. One hundred microliters of bacterial suspension (CV026 in Luria-Bertani (LB)) was added to each well to obtain a final inoculum concentration of 5.2 × 10^7^ CFU mL^−1^ and a working volume of 200 µL. Ofloxacin was used as a positive control for all bacterial cultures. The in vitro bacterial cultures were incubated at 30 °C with constant agitation for 24 h, and the optical density (OD) was monitored at 595 nm using a Bio-Rad iMark microplate absorbance reader version 1.02.01 (Biorad, Hércules, CA, USA).

### 4.5. Violacein Inhibition Assay

The production of violacein pigment by *C. violaceum* in the presence or absence of EOs was quantified spectrophotometrically [56,57]. Briefly, an inoculum of the bacterium was prepared at 0.13 OD (595 nm) and incubated in 3 mL of LB broth supplemented with C6-HSL medium in an Erlenmeyer flask. We added 150 microliters of prepared EO dilutions at different subinhibitory concentrations to each experimental assay. A total of 150 µL of DMSO was used as a control. These flasks were incubated at 30 °C for 20 h and samples from these inhibition assays were withdrawn at different times.

Subsequently, one milliliter of each sample was centrifuged at 13,000× *g* for 10 min. The supernatant was discarded, and the pellet was solubilized in 1 mL of DMSO. The final solution was vortexed for 30 s to homogenize the violacein and centrifuged at 13,000× *g* for 10 min to remove cell debris. Violacein was quantified spectrophotometrically at a wavelength of 595 (UV-1800 Shimadzu, Japan) [58]. The percentage inhibition of violacein production in the presence of oil from *L. origanoides* was determined as follows: [(OD control−OD treated)/OD control] × 100%. Simultaneously, the cell viability of strain CV026 was determined by counting viable bacteria to evidence that cell viability was not reduced at the subinhibitory concentrations evaluated. All experiments were performed with three biological replicates.

### 4.6. Metabolite Extraction from Bacterial Cells

Microbial metabolites were extracted using the method by Zhou and collaborators [59] with some modifications. A bacterial inoculum was prepared at an OD of 0.1 (600 nm) and incubated in 3 mL of LB broth supplemented with C6-HSL in an Erlenmeyer flask. A total of 150 µL of a 0.2 mg/mL concentration of LOT-II EOs was added to each experimental assay. A total of 150 µL of DMSO was used as a control. The flasks were incubated at 28 °C for 24 h. The bacterial cultures were washed with 0.1% peptone water and then centrifuged three times at 5000 rpm for 10 min at 4 °C. Subsequently, 500 µL of aqueous methanol (50% *v*/*v*) was added, followed by acetonitrile (HPLC grade). This sample was sonicated using E30H ultrasound equipment (Elma, Singen, Germany) for 15 min in an ice bath and then centrifuged at 14,000 rpm for 20 min at 4 °C. The obtained supernatant was placed in an Eppendorf tube, and the solvent was evaporated using a Savant Speed Vac SPD120 vacuum concentrator (Thermo Fisher Scientific, Asheville, NC, USA). Finally, the pellet was reconstituted with 400 µL of aqueous methanol (70% *v*/*v*) and stored at −80 °C for further analysis using a UHPLC-Orbitrap-MS. Z-Gly-Tyr-OH was used as an internal standard at a concentration of 5 ppm, and was added at the beginning of metabolite extraction. All experiments were performed in triplicate.

### 4.7. LC/MS Profiling of Metabolites

An aliquot (500 µL) of the sample was analyzed using a UHPLC-ESI-Orbitrap-HRMS. A DionexTM UltimateTM 3000 UHPLC (Thermo Scientific, Sunnyvale, CA, USA), equipped with a degasser (SRD-3400), a binary gradient pump (HPG3400RS), an autosampler (WPS 300TRS), and a thermostated unit for the column (TCC 3000) was used. A Hypersil GOLD ™ aǪ column (Thermo Scientific, Sunnyvale, CA, USA; 100 × 2.1 mm, 1.9 µm particle size) was used under operating conditions of 30 °C. A mixture of 0.2% formic acid in water (A) and 0.2% formic acid in acetonitrile (B) was used as the mobile phase. An initial gradient condition was performed as follows: 100% A changing linearly to 100% B in 8 min, kept constant for 4 min, returned to 100% A in 1 min, and kept in equilibrium for 3 min. A mobile phase of 300 µL/min and an injection volume of 1 µL were used. A UHPLC was coupled to a high-resolution mass spectrometer with an Orbitrap-type ion current detection system (Exactive Plus, Thermo Scientific, Sunnyvale, CA, USA) via a heated electrospray interface (HESI-II) and operated in positive ion mode at 350 °C, a capillary voltage of +3500 V and a temperature of 320 °C. The orbitrap detector was operated in full scan mode (Full MS Scan) with a resolution of 70,000. Positive ions were sent for fragmentation to the HCD (Higher-Energy Collisional Dissociation cell) at different energies (20, 30, 40, and 50 eV) in stepped-scan mode. For each collision energy, an RFWHM resolution of 35,000 was used, using an AGC of 3 × 10^6^ and an injection time in the C-trap chamber of 50 ms. All mass spectra were obtained in the *m*/*z* range of 80–1000 u.m.a. A similar methodology was used for the negative ionization mode.

### 4.8. Data Processing and Statistical Analysis

In this study, nine biological replicates were performed to analyze the effect of *L. origanoides* EO on the metabolic pathways of *C. violaceum* for a total of 18 data points. Data from mass spectrometry analyses were converted from Thermo raw files to mzXML using msconvert from ProteoWizard [60]. The obtained data were analyzed with Thermo Xcalibur v3.1 software (Thermo Scientific, Sunnyvale, CA, USA). For data processing, XCMS (Version 3.7.1) [61] online software was used to perform alignment and deconvolution of data from the control group, treatment, and quality control samples. Subsequently, the data with a coefficient of variation of less than 30% were filtered, and all data were clustered via principal component analysis (PCA) and analyzed with MetaboAnalyst software (Version 5.0) [62]. Normalization was performed according to the total sum of areas, logarithmic transformation, and scaling of the mean centering data for each sample of the control and treated groups. Then, fold change (FC) and the Log2FC were determined. Finally, *p*-values and the FDR (false discovery rate) value were calculated. A metabolite is considered statistically significant if its *p*-value and FDR are less than 0.05; otherwise, it is considered a false discovery [63].

Putative identification of statistically significant metabolites was carried out by comparing the mass/charge ratios (*m*/*z*) determined for each molecule (together with the possible adducts) with those reported in the mass spectrometry database (Kegg, HMDB, LipidMaps, Metlin, NP Atlas, KNApSAcK, mass bank and MINE). Finally, metabolic pathways possibly affected by the alteration of the putative identified metabolites were determined with Metaboanalyst software (Version 5.0). Chemical identification was reported as putatively annotated compounds (level 2 of identification) in accordance with the Metabolomics Standards Initiative (MSI) [64,65].

### 4.9. Statistical Analysis

All results are expressed as the means and their respective standard deviations for each of the assays. All statistical analyses were performed on the R platform, and *p* < 0.05 was considered statistically significant. Significant changes are indicated by asterisks in the figures.

## 5. Conclusions

Essential oils represent promising control agents to be used in therapeutic treatments. Based on our experimental results, we concluded that EO from *Lippia origanoides* not only affected violacein production in *C. violaceum,* but also inhibited biological functions related to amino acid metabolism, specifically arginine and nucleotide biosynthesis, showing possible effects at the levels of protein synthesis, the mechanisms of pathogenicity, and the mechanisms of biofilm formation—all without affecting the viability of the microorganism. These results provide new insight into the possible underlying QS-inhibitory mechanism of EOs and deepen our understanding of the response of CV026 to EO from LOT-II. However, further studies are needed to determine the safety and effectiveness of LOT-II EO in in vivo experiments and clinical trials before it can be considered as a novel therapeutic agent.

## Figures and Tables

**Table 1:** Major chemical compounds present in the EOs assessed. The relative amount of each metabolite is reported as a percentage (%).

Plant Species	Botanical Family	Voucher Number	Code	Essential Oil Yield (%, *w*/*w*)		Main Compounds
				MWHD	SD	
*Steiractinia aspera* Cuatrec	Asteraceae	20891 Herbarium UIS, Bucaramanga	SA	0.1	-	α-Pinene (24.9%), β-pinene (14.8%), germacrene D (13.1%), β-phellandrene (10.1%), α-phellandrene (6.3%), sabinene (4.6%), *p*-cymene (4.5%), trans-β-caryophyllene (3.1%), α-copaene (2.6%), and limonene (2.4%).
*Turnera diffusa* Willd. ex Schult	Passifloraceae	22032 Herbarium UIS, Bucaramanga	TD-I	-	0.3	Dehydrofukinone (25.4%), aristolochene (17.9%), valencene (7.4%), β-selinene (5.2%), trans-β-caryophyllene (4.0%), β-elemene (4.0%), premnaspirodiene (3.7%), guaiol (3.5%), germacra-4,5,10-trien-1-α-ol (3.5%), and caryophyllene oxide (3.2%).
*Calycolpus moritzianus* (O. Berg) Burret	Myrtaceae	21982 Herbarium UIS, Bucaramanga	CM-I	-	0.2	1,8-Cineole (19.1%), limonene (17.6%), trans-β-caryophyllene (6.3%), viridiflorol (5.7%), α-pinene (5.1%), trans, trans-geranyl-linalool (4.0%), trans-nerolidol (3.5%), α-copaene (3.2%), selina-3,7 (11)-diene (2.8%), and viridiflorene (2.7%).
*Piper aduncum* L.	Piperaceae	22033 Herbarium UIS, Bucaramanga	PA	-	0.4	Piperitone (14.8%), trans-β-caryophyllene (7.4%), viridiflorol (6.5%), limonene (6.0%), δ-cadinene (5.5%), α-pinene (4.6%), α-phellandrene (4.4%), caryophyllene oxide (3.8%), 1,8-cineole (3.6%), and *p*-cymene (3.0%).
*Elaphandra quinquenervis* (S.F. Blake) H. Rob	Asteraceae	COL 587094 Herbarium UN, Bogotá	EQ	0.2	-	b-Pinene (20.7%), germacrene D (20.7%), sabinene (9.7%), α-pinene (6.8%), trans-β-caryophyllene (5.1%), limonene (4.5%), β-cubebene (3.5%), α-humulene (2.6%), premnaspirodiene (2.6%), δ-cadinene (2.6%), and α-phellandrene (2.4%).
*Hyptis dilatate* Benth	Lamiaceae	22187 Herbarium UIS, Bucaramanga	HD	0.8	-	trans-β-Caryophyllene (20.2%), camphor (16.1%), ∆^3^-carene (15.5%), α-pinene (10.5%), palustrol (8.7%), α-gurjunene (4.7%), ledol (3.4%), limonene (2.4%), camphene (1.7%), viridiflorene (1.5%), and aromadendrene (1.5%).
*Satureja viminea* L.	Lamiaceae	COL 566449 Herbarium UN, Bogotá	SV	-	0.6	*p*-Menth-3-en-8-ol (32.4%), pulegone (16.1%), trans-9-epi-caryophyllene (8.9%), trans-β-caryophyllene (8.4%), caryophyllene oxide (4.3%), spathulenol (3.6%), benzyl benzoate (2.4%), δ-cadinene (2.2%), pulegol (1.8%), and *p*-mentha-3,8-diene (1.5%).
*Psidium sartorianum* (O. Berg) Burret	Myrtaceae	COL 578359 Herbarium UN, Bogotá	PS	-	0.5	trans-β-Caryophyllene (12.7%), caryophyllene oxide (12.0%), dehydrofukinone (7.5%), cariophylla-4(12),8(13)-dien-5-β-ol (4.8%), germacrene B (4.1%), 1,8-cineole (3.7%), *p*-cymene (2.9%), β-pinene (2.7%), selina-3,7(11)-diene (2.5%), β-selinene (2.1%), and premnaspirodiene (2.0%).
*Varronia curassavica* Jacq.	Boraginaceae	22038 Herbarium UIS, Bucaramanga	VC	-	0.2	trans-β-Caryophyllene (19.2%), germacrene D (12.3%), trans-β-guaiene (11.8%), α-pinene (9.4%), α-copaene (7.0%), β-pinene (4.1%), germacrene D (3.9%), β-elemene (2.8%), δ-cadinene (2.8%), and α-humulene (2.7%).
*Ocimum basilicum* L.	Lamiaceae	22227 Herbarium UIS, Bucaramanga	OB	-	0.2	Linalool (42.7%), estragole (18.6%), 1,8-cineole (8.1%), germacrene D (4.9%), epi-γ-cadinol (4.2%), γ-cadinene (3.7%), α-humulene (2.5%), β-elemene (2.2%), byciclogermacrene (2.2%), and trans-α-bergamotene (1.1%).
*Lippia origanoides* Kunth (thymol chemotype)	Verbenaceae	22189 Herbarium UIS, Bucaramanga	LOT-II	-	0.7	Thymol (71.7%), *p*-cymene (10.5%), carvacrol (4.4%), β-myrcene (2.1%), γ-terpinene (2.0%), caryophyllene oxide (1.6%), thymyl methyl ether (0.9%), trans β-caryophyllene (0.9%), humulene epoxide II (0.7%), and terpinen-4-ol (0.7%).
*Lippia micromera* Schauer	Verbenaceae	22190 Herbarium UIS, Bucaramanga	LM	0.6	-	*p*-Cymene (26.8%), thymyl methyl ether (26.3%), thymol (17.8%), thymyl acetate (5.7%), γ-terpinene (5.4%), 1,8-cineole (5.1%), α-terpinene (2.0%), β-myrcene (2.0%), trans-β-caryophyllene (1.7%), α-thujene (1.3%), and caryophyllene oxide (0.9%).
*Piper reticulatum* L.	Piperaceae	21969 Herbarium UIS, Bucaramanga	PR	0.1	-	Germacrene D (14.5%), β-eudesmol (9.2%), β-elemene (7.4%), trans-β-caryophyllene (7.4%), germacrene B (4.9%), trans-nerolidol (4.9%), linalool (4.8%), β-selinene (2.9%), bicyclogermacrene (2.5%), and ishwarane (2.1%).
*Ageratina popayanensis* (Hieron) R. King and H. Rob	Asteraceae	21975 Herbarium UIS, Bucaramanga	AP-I	0.3	-	α-Pinene (27.0%), camphene (11.4%), α-phellandrene (10.5%), β-pinene (8.4%), limonene (7.0%), *p*-cymene (4.7%), trans-verbenol (4.0%), trans-β-caryophyllene (3.1%), β-myrcene (2.2%), and verbenone (2.0%).
*Ocimum campechianum* Mill.	Lamiaceae	20889 Herbarium UIS, Bucaramanga	OC	0.4	-	Eugenol (35.3%), 1,8-cineole (15.6%), β-selinene (11.0.%), trans-β-caryophyllene (7.4%), germacrene D (5.6%), α-selinene (4.8%), β-pinene (2.4%), β-elemene (1.9%), and α-humulene (1.5%).

UIS: Industrial University of Santander (Bucaramanga, Colombia). MWHD: microwave-assisted hydrodistillation. SD: steam distillation.

**Table 2:** Minimal inhibitory concentration to inhibit 50% of the bacterial population (MIC_50_) and minimal bactericidal concentration (MBC) (mg/mL) determined for the EOs assessed. Values are means ± SDs of triplicate determinations. Different letters (a, b and c) indicate significant differences between the tested groups.

Essential Oil	CV026 MIC_50_/MBC
*Steiractinia aspera*	>3/>3
*Turnera diffusa*	0.75 ± 0.14 ^b^/1.5 ± 0.09 ^b^
*Calycolpus moritzianus*	3.0 ± 0.11 ^a^/>3.0
*Piper aduncum*	1.0 ± 0.13 ^b^/3 ± 0.21 ^a^
*Elephandra qinquenervis*	>3.0/>3.0
*Hyptis dilatata*	0.75 ± 0.08 ^b^/1.5 ± 0.12 ^a^
*Satureja viminea*	1.5 ± 0.12 ^c^/3 ± 0.27 ^c^
*Psidium sartorianum*	3.0 ± 0.18 ^a^/>3.0
*Varronia curassavica*	0.5 ± 0.06 ^a^/0.75 ± 0.15 ^c^
*Ocimum basilicum*	0.37 ± 0.04 ^a^/0.75 ± 0.06 ^a^
*Lippia origanoides*	0.37 ± 0.09 ^a^/0.5 ± 0.14 ^b^
*L. micromera*	1.5 ± 0.13 ^b^/3 ± 0.21 ^b^
*P. reticulatum*	0.75 ± 0.17 ^c^/1.5 ± 0.18 ^b^
*Ageratina popayanensis*	3.0 ± 0.13 ^b^/>3.0
*O. campechianum*	3.0 ± 0.26 ^a^/>3.0

**Table 3:** Different putative metabolites identified in *C. violaceum* CV026.

Metabolites Identified	(ESI) Mode	Formula	Mass Error (Δppm)	*p*-Value	VIP	Log2FC
Violacein	+	C_20_H_13_N_3_O_3_	0.0843	4.77 × 10^−14^	33.960	13.941
Adenosine	+	C_10_H_13_N_5_O_4_	0.0149	1.91 × 10^−3^	32.488	−32.848
Deoxyviolacein	+	C_20_H_13_N_3_O_2_	0.4940	4.98 × 10^−6^	32.076	26.050
Succinyl arginine	+	C_10_H_18_N_4_O_5_	0.5718	6.03 × 10^−5^	31.507	−17.638
Saccharopine	+	C_11_H_20_N_2_O_6_	11.743	6.69 × 10^−12^	21.417	−51.693
Phenylalanine	+	C_9_H_11_NO_2_	20.379	2.62 × 10^−11^	25.167	−31.323
Hydroxy trimethyl lysine	+	C_9_H_21_N_2_O_3_	0.8038	1.76 × 10^−8^	29.245	17.621
Guanine	+	C_5_H_6_N_5_O	0.4015	4.99 × 10^−9^	28.943	−24.310
Adenine	+	C_5_H_5_N_5_	11.942	1.78 × 10^−18^	28.725	−27.750
Acetyllysine	+	C_8_H_16_N_2_O_3_	0.9895	5.78 × 10^−4^	27.820	−24.670
Tryptophan	+	C_11_H_12_N_2_O_2_	0.8116	1.08 × 10^−4^	27.439	−23.324
Nitrobenzene	+	C_6_H_5_NO_2_	0.7515	3.29 × 10^−10^	27.090	10.349
β-Citryl glutamic acid	+	C_11_H_15_NO_10_	12.256	1.37 × 10^−11^	26.703	34.022
Xanthine	+	C_5_H_4_N_4_O_2_	0.2436	2.62 × 10^−11^	26.893	−19.523
3-Sulfomuconate	+	C_6_H_6_O_7_S	21.621	1.11 × 10^−5^	26.133	22.631
Acetyl spermidine	+	C_9_H_21_N_3_O	0.4366	6.39 × 10^−10^	25.537	13.183
Uridine	+	C_9_H_12_N_2_O_6_	0.8839	8.94 × 10^−11^	25.420	−16.253
9-Octadecenamide	+	C_18_H_35_NO	0.2269	5.18 × 10^−10^	24.482	−14.532
3-Indolebutyric acid	+	C_12_H_13_N_2_O_3_	11.481	7.28 × 10^−12^	24.146	−18.690
Arginine	+	C_6_H_14_N_4_O_2_	14.242	1.27 × 10^−10^	24.066	−62.861
Acetyl cadaverine	+	C_7_H_16_NO_2_	0.7951	1.35 × 10^−5^	25.148	21.813
Citidine	+	C_9_H_13_N_3_O_5_	16.728	7.71 × 10^−4^	25.476	−26.352
2-Deoxycytidine	+	C_9_H_13_N_3_O_4_	0.5804	1.27 × 10^−10^	23.738	−30.419
Valyl aspartic acid	+	C_9_H_16_N_5_O_2_	0.8803	2.62 × 10^−11^	23.305	10.956
Suberylglycine	+	C_10_H_17_NO_5_	0.8370	2.67 × 10^−9^	22.748	10.932
Linatine	+	C_10_H_17_N_3_O_5_	29.349	2.24 × 10^−15^	22.531	−21.927
Ethylthymine	+	C_7_H_10_N_2_O_2_	0.5456	4.39 × 10^−6^	22.409	−23.307
Isoleucylvaline	+	C_11_H_22_N_2_O_3_	0.8311	7.99 × 10^−8^	22.076	−20.990
Gly-Asn-Asn	+	C_10_H_17_N_5_O_6_	13.479	1.01 × 10^−12^	21.509	−21.012
Citrulline	+	C_6_H_13_N_3_O_3_	0.9092	8.58 × 10^−4^	23.977	17.810
Actinamine	+	C_8_H_18_N_2_O_4_	0.6729	4.37 × 10^−7^	21.186	−12.726
γ-Glutamyl glutamate	+	C_10_H_16_N_3_O_6_	12.383	4.07 × 10^−8^	21.168	12.839
4-Dimethylamine phenylalanine	+	C_11_H_16_N_2_O_2_	0.7831	5.60 × 10^−8^	20.847	17.459
C_16_ Sphinganine	+	C_16_H_35_NO_2_	0.3016	8.64 × 10^−14^	20.828	10.338
Dimethylpentadiene	+	C_7_H_12_	0.8938	2.83 × 10^−7^	20.589	−26.026
Pentadecanoyl glycine	+	C_17_H_33_NO_3_	15.648	1.19 × 10^−6^	20.236	13.863
Leucyl-glutamate	-	C_11_H_20_N_2_O_5_	0.9031	1.78 × 10^−10^	49.300	−35.170
Ethyl pyruvate	-	C_5_H_8_O_3_	13.483	8.27 × 10^−8^	41.568	−52.600
4-Pyridoxic acid	-	C_8_H_9_NO_4_	0.7120	1,01 × 10^−1^	39.932	57.321
Phosphoric acid	-	H_3_O_4_P	0.3490	2.62 × 10^−11^	32.446	−31.130
Pyridoxal	-	C_8_H_9_NO_3_	0.8944	6.69 × 10^−11^	32.358	−27.880
3-Methylxanthine	-	C_6_H_6_N_4_O_2_	0.5032	1.46 × 10^−5^	30.088	−24.965
Myristic acid	-	C_14_H_28_O_2_	13.498	5.22 × 10^−9^	27.739	27.619
Ophthalmic acid	-	C_11_H_19_N_3_O_6_	0.7217	1.58 × 10^−7^	26.873	−23.755
Pantoic acid	-	C_6_H_11_O_4_	0.8352	7.46 × 10^−12^	25.544	−10.324
Phe-His-Gln	-	C_20_H_26_N_6_O_5_	14.503	7.89 × 10^−12^	24.931	−20.269
3-Hydroxytetradecanoic acid	-	C_14_H_28_O_3_	10.338	5.06 × 10^−6^	24.424	24.480
γ-Glutamyl ornithine	-	C_10_H_19_N_3_O_5_	0.6948	3.69 × 10^−12^	23.986	−36.600
Isoleucyl-glutamate	-	C_11_H_20_N_2_O_5_	17.221	1.24 × 10^−7^	23.800	−64.377
2-Oxoglutaric acid	-	C_5_H_6_O_5_	14.721	1.32 × 10^−9^	22.360	26.076
Leu-Val-Trp	-	C_22_H_32_N_4_O_4_	21.655	2.75 × 10^−5^	19.900	34.787
Lysopine	-	C_9_H_18_N_2_O_4_	0.6772	2.16 × 10^−10^	18.651	14.420
Anthranilic acid	-	C_7_H_7_NO_2_	0.1621	5.57 × 10^−5^	28.607	−27.437
Ornithine	-	C_5_H_12_N_2_O_2_	18.575	2.89 × 10^−12^	25.407	23.045
Hypoxanthine	-	C_5_H_4_N_4_O	0.7997	4.68 × 10^−9^	21.823	−21.632

(ESI) mode: electrospray ionization acquisition mode. VIP: variable importance in projection. Log2FC: logarithm of fold change ratios.

**Table 4:** Main metabolic pathways affected by *L. origanoides* EO in the inhibition of QS in *C. violaceum*.

Metabolic Pathway	Metabolites
Tryptophan metabolism	Anthranilic acid, tryptophan, deoxyviolacein and violacein
Nucleotide biosynthesis	Adenine, adenosine, guanine, 3-methylxanthine, xanthine, ethylthymine, cytidine, 2-deoxycytidine, uridine, and hypoxanthine
Arginine metabolism	Succinylarginine, ornithine, acetylspermidine, Arginine and citrulline
Vitamin biosynthesis	Pyridoxal, pantoic acid and 4-pyridoxic acid
Lysine metabolism	Saccharopine, hydroxy-trimethyl lysine and acetyllysine
Peptides	Valylaspartic acid, isoleucylvaline, Gly-Asn-Asn, γ-Glutamyl glutamate, Leucyl-Glutamate, Phe-His-Gln, γ-Glutamyl ornithine, isoleucyl-glutamate, and Leu-Val-Trp
Organic acids	Ethyl pyruvate, phosphoric acid, 3-hydroxytetradecanoic acid and 2-oxoglutaric acid
Lipids	C16 Sphinganine and myristic acid
Phenylalanine metabolism	Phenylalanine and 4-dimethylamine phenylalanine
Others	Nitrobenzene, β-citryl glutamic acid, 3-sulfomuconate, 9-octadecenamide, 3-indolebutyric acid, acetylcadaverine, Suberylglycine, linatine, actinamine, dimethylpentadiene, pentadecanoyl glycine, ophthalmic acid, and lysopine

## Data Availability

Not applicable.

## Supplementary Materials

The following supporting information can be downloaded at: https://www.mdpi.com/article/10.3390/antibiotics12050814/s1, Table S1. Linear retention indices of the main essential oil components. Table S2. Evaluation of the anti-QS activity of EOs against CV026. Table S3. Colony-forming units/mL of CV026 bacterial strain measured in the control and treated CV cells (with *L. origanoides* EO). Figure S1. Aromatic plant cultivation plots at the CENIVAM research center (Industrial University of Santander, Bucaramanga, Colombia). Figure S2. Chromatographic profiles of essential oils distilled from 15 aromatic plants cultivated at the CENIVAM research center (Industrial University of Santander, Bucaramanga, Colombia). GC/MS, electron ionization (EI, 70 eV). Split 30:1. References [55,66,67] are cited in the Supplementary Materials.
