# Supplementary material for: Metabolomic Analysis of the Effect of Lippia origanoides Essential Oil on the Inhibition of Quorum Sensing in Chromobacterium violaceum
Source: Antibiotics (Basel). 2023 Apr 26;12(5):814. doi: 10.3390/antibiotics12050814 (PMC10215488; doi:10.3390/antibiotics12050814)
Supplement: Supplementary file 1 [file antibiotics-12-00814-s001.zip › antibiotics-2334358-Supplementary material-Table.pdf]

Table S1. Linear retention indices of the main essential oil components.

Compound	Linear retention indices				Identification criteria
	Polar column*		Non-polar column**		
	Exp.	Lit.	Exp.	Lit.	
α-Thujene	929	924 [1]	1026	1027 [2]	a, b
α-Pinene	937	936 [2]	1022	1025 [2]	a, b, c
Camphene	953	950 [2]	1067	1068 [2]	a, b, c
Sabinene	976	973 [2]	1120	1122 [2]	a, b, c
β-Pinene	980	978 [2]	1108	1110 [2]	a, b
β-Myrcene	990	989 [2]	1160	1161 [2]	a, b, c
α-Phellandrene	1008	1004 [2]	1164	1168 [2]	a, b, c
Δ^3-Carene	1012	1011 [2]	1146	1147 [2]	a, b
α-Terpinene	1021	1017 [2]	1177	1178 [2]	a, b, c
p-Cymene	1026	1024 [2]	1268	1270 [2]	a, b, c
Limonene	1032	1030 [2]	1197	1198 [2]	a, b, c
β-Phellandrene	1036	1030 [2]	1205	1209 [2]	a, b
1,8-Cineole	1037	1032 [2]	1210	1211 [2]	a, b, c
γ-Terpinene	1062	1060 [2]	1243	1245 [2]	a, b, c
p-Mentha-3,8-diene	1073	1068 [1]	1264	1271 [3]	a, b
Linalool	1103	1099 [2]	1543	1543 [2]	a, b, c
trans-Verbenol	1150	1144 [2]	1678	1680 [2]	a, b
Camphor	1156	1143 [2]	1519	1515 [2]	a, b
p-Menth-3-en-8-ol	1156	1145 [1]	1611	1600 [3]	a, b
Terpinen-4-ol	1187	1177 [2]	1608	1601 [2]	a, b, c
Estragole	1203	1196 [2]	1665	1671 [2]	a, b, c
Verbenone	1213	1206 [2]	1708	1721 [2]	a, b
trans-Pulegol	1216	1213 [1]	1722	-	a, b
Thymyl methyl ether	1234	1234 [2]	1590	1587 [2]	a, b
Pulegone	1245	1234 [2]	1651	1654 [2]	a, b
Piperitone	1264	1264 [3]	1738	1730 [2]	a, b
Thymol	1292	1290 [2]	2165	2164 [2]	a, b, c
Carvacrol	1302	1300 [2]	2192	2211 [2]	a, b, c
Thymyl acetate	1348	1356 [2]	1860	1867 [2]	a, b
Eugenol	1365	1358 [2]	2171	2163 [2]	a, b, c
α-Copaene	1387	1376 [2]	1497	1491 [2]	a, b
β-Cubebene	1396	1387 [1]	1543	1542 [2]	a, b
β-Elemene	1396	1390 [2]	1592	1591 [2]	a, b
α-Gurjunene	1419	1409 [1]	1536	1529 [2]	a, b

Compound	Linear retention indices				Identification criteria
	Polar column*		Non-polar column**		
	Exp.	Lit.	Exp.	Lit.	
trans-β-Caryophyllene	1432	1420 [2]	1600	1598 [2]	a, b, c
Aromadendrene	1452	1441 [2]	1614	1620 [2]	a, b, c
α-Humulene	1469	1468 [3]	1674	1667 [2]	a, b, c
Aristolochene	1483	1487 [1]	1687	1697 [4]	a, b
Ishwarane	1486	1465 [1]	1653	1636 [3]	a, b
Germacrene D	1494	1481 [2]	1710	1708 [2]	a, b, c
Premnaspirodiene	1501	1505 [1]	1725	-	a, b
β-Selinene	1502	1489 [1]	1722	1717 [2]	a, b
Viridiflorene	1502	1496 [1]	1700	1696 [2]	a, b
Valencene	1507	1496 [1]	1713	1729 [2]	a, b
Bicyclogermacrene	1509	1500 [1]	1735	1734 [2]	a, b
α-Selinene	1509	1498 [1]	1737	1725 [2]	a,b
trans-β-Guaiene	1517	1502 [1]	-	-	a, b
γ-Cadinene	1525	1513 [1]	1763	1763 [2]	a, b
δ-Cadinene	1526	1523 [2]	1754	1756 [2]	a, b
Selina-3,7(11)-diene	1550	1545 [1]	1780	1783 [2]	a, b
trans-Nerolidol	1564	1561 [1]	2036	2036 [2]	a, b, c
Germacrene B	1574	1559 [1]	1828	1824 [2]	a, b
Palustrol	1586	1567 [1]	1930	1930 [2]	a, b
Spathulenol	1590	1577 [1]	2116	2127 [2]	a, b
Caryophyllene oxide	1596	1582 [1]	1981	1986 [2]	a, b, c
Guaiol	1606	1600 [1]	2085	2089 [2]	a, b, c
Viridiflorol	1611	1592 [1]	2080	2090 [2]	a, b
Ledol	1621	1612 [3]	2026	2039 [2]	a, b
Humulene epoxide II	1627	1608 [1]	2036	2047 [2]	a, b
Caryophylla-4(12),8(13)-dien-5-β-ol	1653	1639 [1]	2287	-	a, b
β-Eudesmol	1665	1650 [2]	2224	2238 [2]	a, b, c
Germacra-4,5,10-trien-1-α-ol	1680	1685 [1]	2365	-	a, b
Benzyl benzoate	1777	1761 [2]	2652	2652 [3]	a, b, c
Dehydrofukinone	1827	1808 [3]	2448	2404 [3]	a, b
trans, trans-Geranyl-linalool	2027	2026 [1]	-	2551 [3]	a, b

Compounds listed in order of elution from the non-polar column.

Exp. – Experimental

Lit. - Literature.

*DB-5MS column. ** DB-WAX column.

Identification criteria:

a - Tentative identification based on linear retention indices
b - Tentative identification based on mass spectra (EI, 70 eV)
c - Confirmatory identification based on the use of standard compounds.

References:

[55]. V. I. Babushok, P. J. Linstrom and I. G. Zenkevich (2011) Retention Indices for Frequently Reported Compounds of Plant Essential Oils, J. Phys. Chem. Ref. Data, 40, 043101. https://doi.org/10.1063/1.3653552.

[66] R. P. Adams, (2007) Identification of essential oil components by gas chromatography/mass spectrometry. 4th ed., Allured Publ., Carol Stream, Illinois, EE. UU.,1-809. https://doi.org/10.1016/j.jasms.2005.07.008.

[67] W. Wallace, (2017) NIST Standard Reference Database 1A. Version 2.3

Table S2. Evaluation of the anti-QS activity of EOs against CV026

Essential oil	MQSIC (mg/mL)	Violacein Inhibition (%)
Steiractinia aspera	3	17,6
Turnera diffusa	0,37	45,8
Calycolpus moritzianus	1,5	23,0
Piper aduncum	0,5	54,6
Elephandra qinquenervis	3	11,5
Hyptis dilatata	0,37	53,2
Satureja viminea	0,75	32,2
Psidium sartorianum	1,5	28,7
Ocimum campechianum	1,5	34,6
Ocimum basilicum	0,18	85,2
Lippia origanoides	0,15	94,0
Lippia micromera	0,75	42,4
Piper reticulatum	0,37	32,5
Ageratina popayanensis	1,5	38,3
Varronia curassavica	0,25	72,1

MQSIC: Minimal Quorum Sensing Inhibitory Concentration

Table S3. Colony-forming units/mL of CV026 bacterial strain measured in the control and treated CV cells (with *L. origanoides* EO)

Time (h)	CFU/mL	
	Control	Treated EO
0	$7.5*10^6 \pm 4$	$7.6*10^6 \pm 2$
24	$9*10^6 \pm 3$	$9.2*10^6 \pm 3$
