# Supplementary material for: Metabolomic Analysis of the Effect of Lippia origanoides Essential Oil on the Inhibition of Quorum Sensing in Chromobacterium violaceum
Source: Antibiotics (Basel). 2023 Apr 26;12(5):814. doi: 10.3390/antibiotics12050814 (PMC10215488; doi:10.3390/antibiotics12050814)
Supplement: Supplementary file 1 [file antibiotics-12-00814-s001.zip › antibiotics-2334358-supplementary-Figure.pdf]

Supplementary Material

Figure S1.

A. Aromatic plant cultivation plots at the CENIVAM research centre (Industrial University of Santander, Bucaramanga, Colombia).

B. Distillation facilities (one of the stainless-steel alembic used in research projects).

C. Lippia origanoides (Verbenaceae family) plant.

D. Satureja viminea (Lamiaceae family) plant.

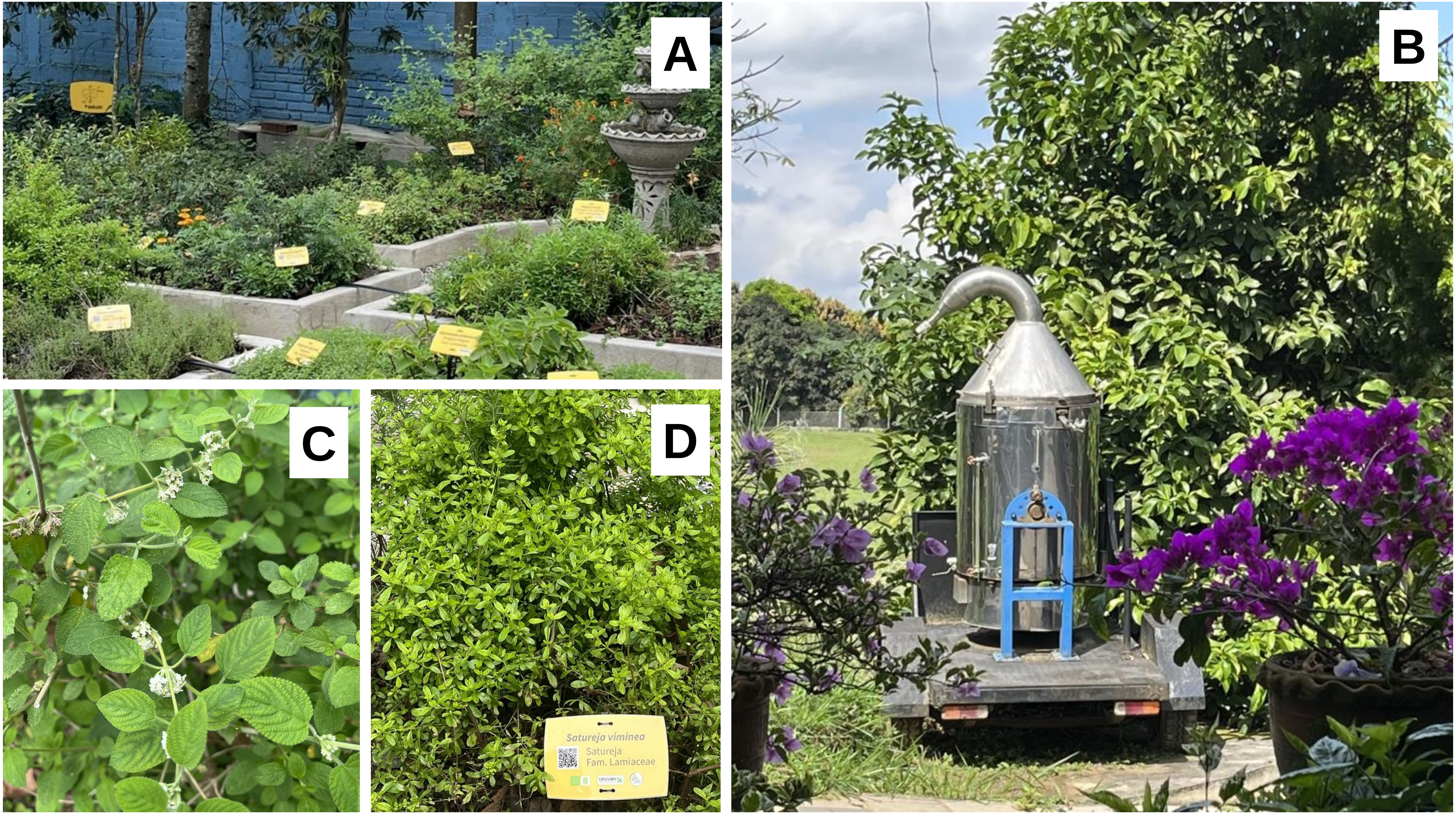
A
B
C
D
Satureja viminea
Satureja
Fam. Lamiaceae

Supplementary Material

Figure S2.

Chromatographic profiles of essential oils distilled from 15 aromatic plants cultivated at the CENIVAM research centre (Industrial University of Santander, Bucaramanga, Colombia). GC/MS, electron ionization (EI, 70 eV). Split 30:1.

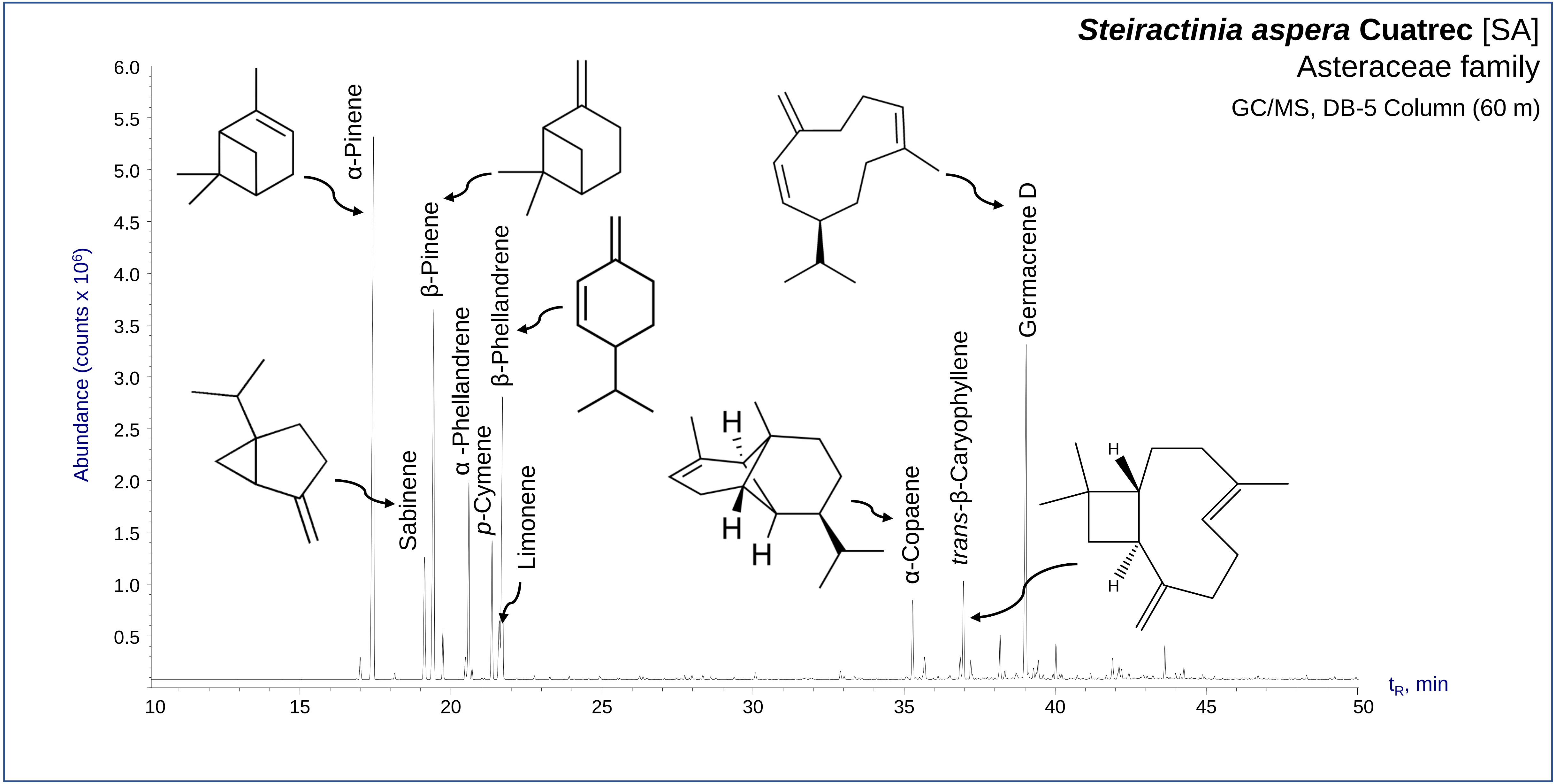

Steiractinia aspera Cuatrec [SA]
Asteraceae family
GC/MS, DB-5 Column (60 m)
Abundance (counts x 10^6)
6.0
5.5
5.0
4.5
4.0
3.5
3.0
2.5
2.0
1.5
1.0
0.5
α-Pinene
β-Pinene
Sabinene
α -Phellandrene
p-Cymene
β-Phellandrene
Limonene
Germacrene D
α-Copaene
trans-β-Caryophyllene
H
H
H
H
H
10
15
20
25
30
35
40
45
50
tR, min

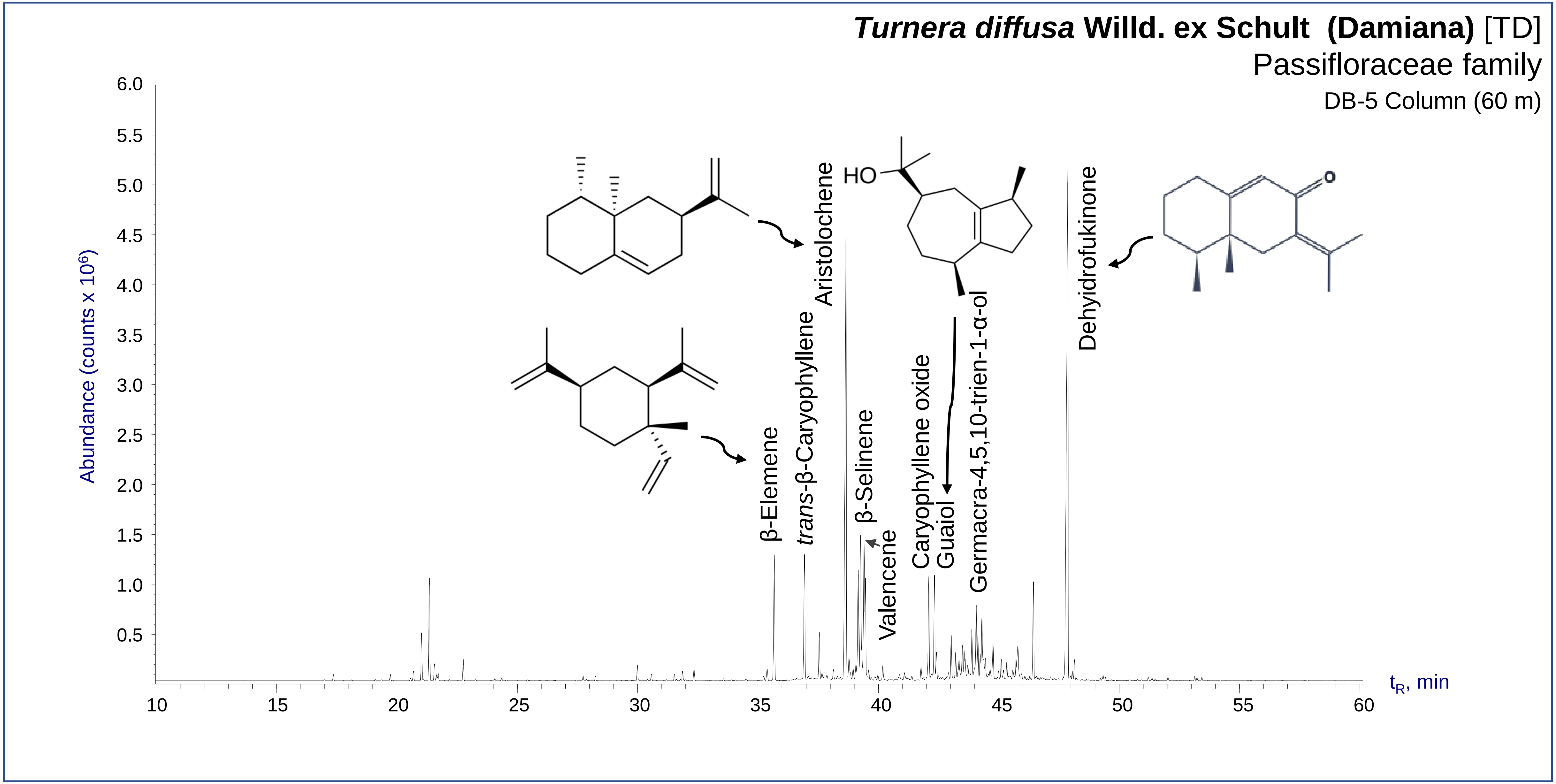

Turnera diffusa **Willd. ex Schult (Damiana)** [TD]
Passifloraceae family
DB-5 Column (60 m)
Abundance (counts x 10⁶)
6.0
5.5
5.0
4.5
4.0
3.5
3.0
2.5
2.0
1.5
1.0
0.5
β-Elemene
trans-β-Caryophyllene
Aristolochene
β-Selinene
Valencene
Caryophyllene oxide
Guaiol
Germacra-4,5,10-trien-1-α-ol
Dehyidrofukinone
HO
O
10
15
20
25
30
35
40
45
50
55
60
tR, min

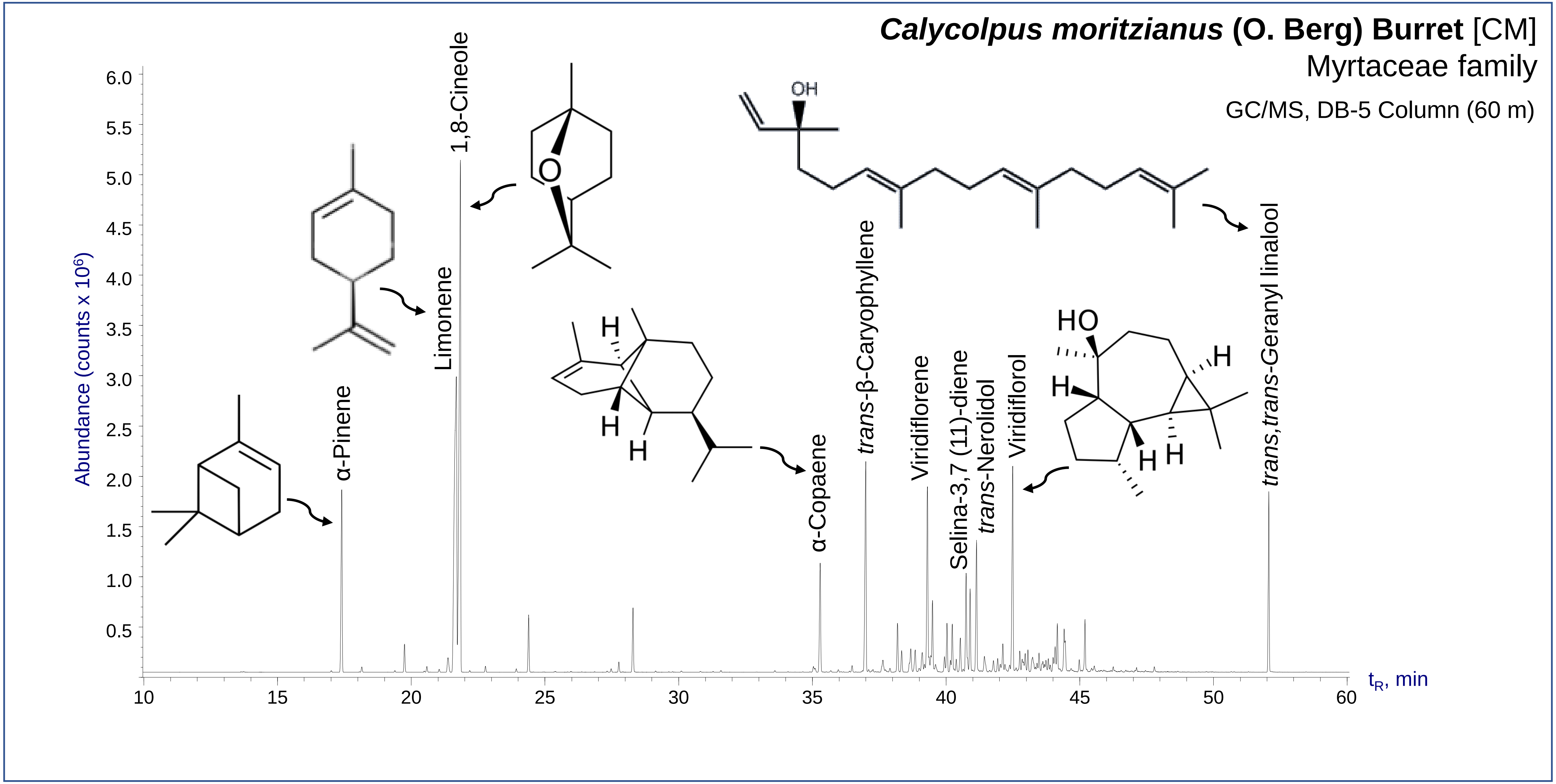

Calycolpus moritzianus (O. Berg) Burret [CM]
Myrtaceae family
GC/MS, DB-5 Column (60 m)
Abundance (counts x 10⁶)
6.0
5.5
5.0
4.5
4.0
3.5
3.0
2.5
2.0
1.5
1.0
0.5
10
15
20
25
30
35
40
45
50
60
tR, min
α-Pinene
Limonene
1,8-Cineole
α-Copaene
trans-β-Caryophyllene
Viridiflorene
Selina-3,7 (11)-diene
trans-Nerolidol
Viridiflorol
trans,trans-Geranyl linalool
OH
O
H
HO

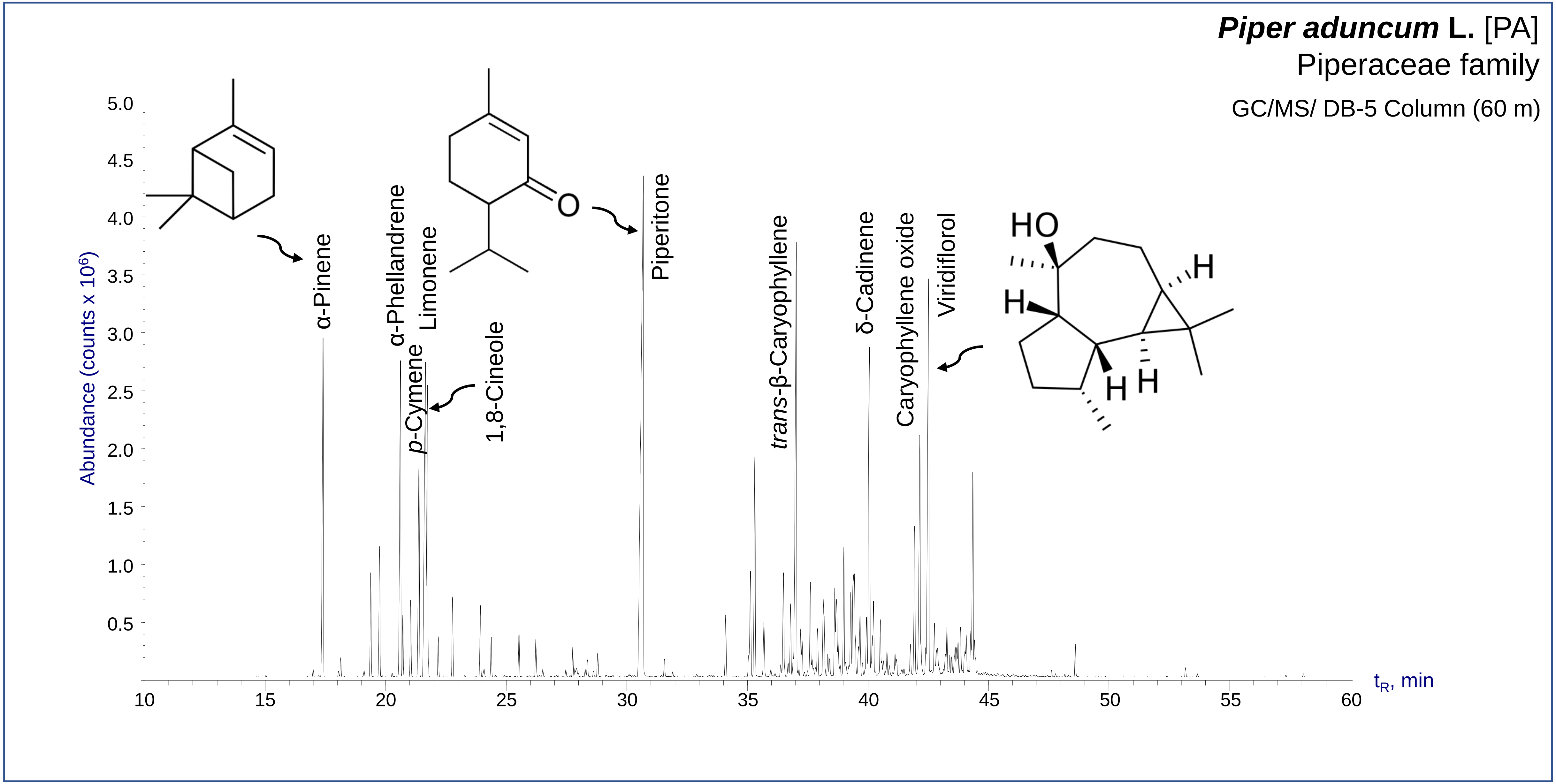

***Piper aduncum* L.** [PA]
Piperaceae family
GC/MS/ DB-5 Column (60 m)
Abundance (counts x 10^6)
5.0
4.5
4.0
3.5
3.0
2.5
2.0
1.5
1.0
0.5
α-Pinene
α-Phellandrene
Limonene
p-Cymene
1,8-Cineole
O
Piperitone
trans-β-Caryophyllene
δ-Cadinene
Caryophyllene oxide
Viridiflorol
HO
H
10
15
20
25
30
35
40
45
50
55
60
t_R, min

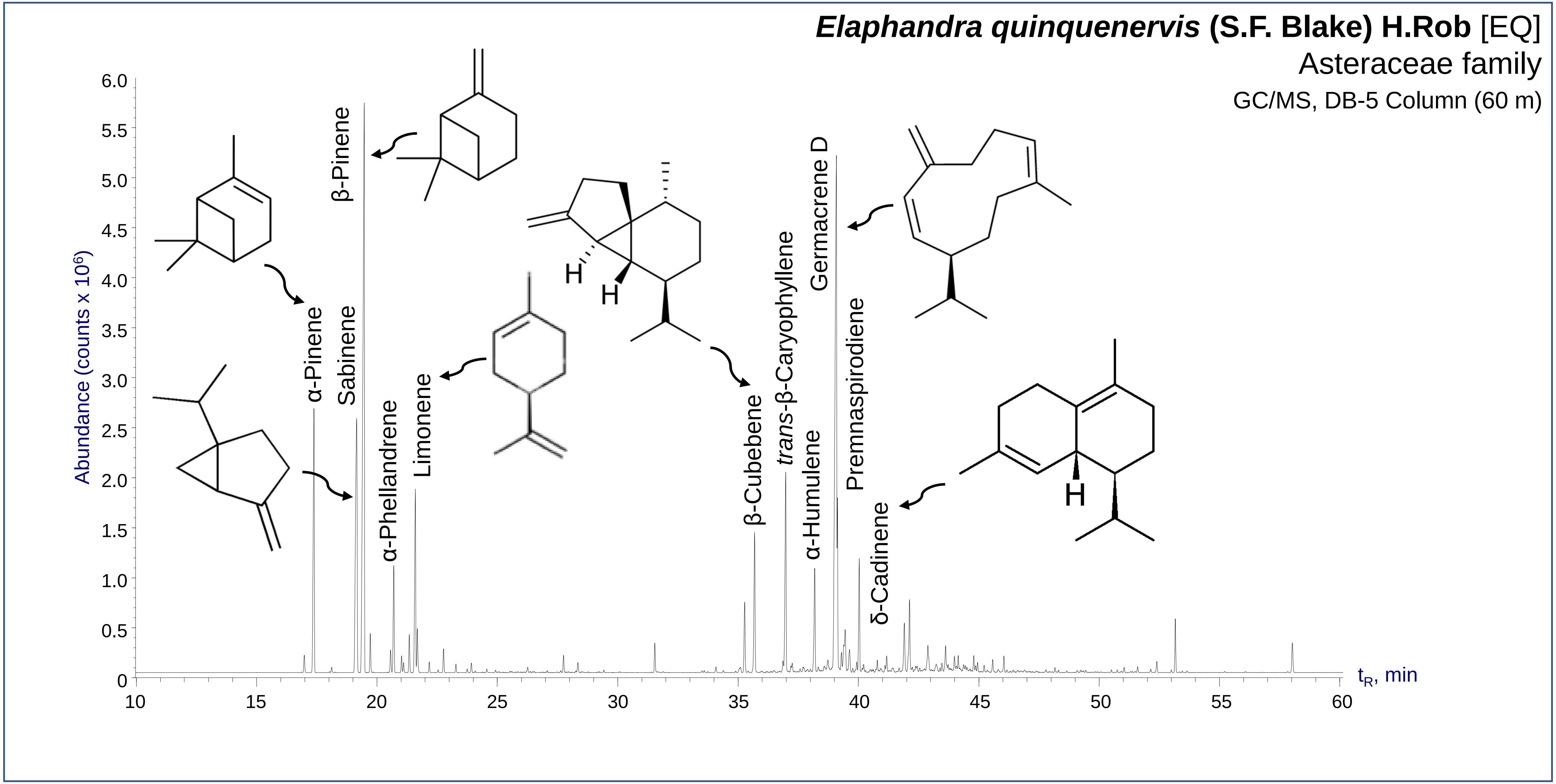

Elaphandra quinquenervis (S.F. Blake) H.Rob [EQ]
Asteraceae family
GC/MS, DB-5 Column (60 m)
Abundance (counts x 10⁶)
6.0
5.5
5.0
4.5
4.0
3.5
3.0
2.5
2.0
1.5
1.0
0.5
0
10
15
20
25
30
35
40
45
50
55
60
t_R, min
α-Pinene
Sabinene
β-Pinene
α-Phellandrene
Limonene
β-Cubebene
trans-β-Caryophyllene
α-Humulene
Germacrene D
Premnaspirodiene
δ-Cadinene
H
H
H

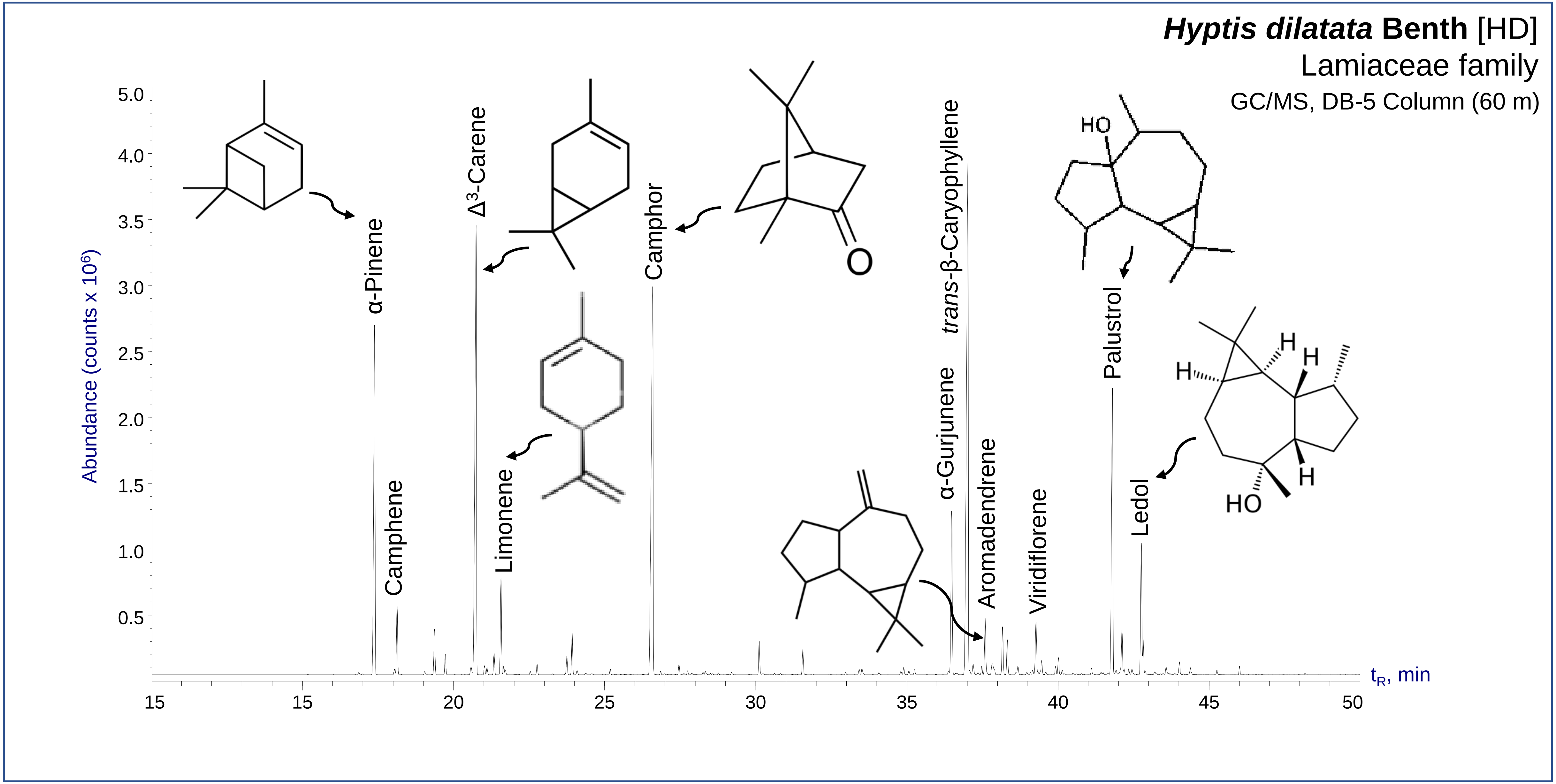

Hyptis dilatata Benth [HD]
Lamiaceae family
GC/MS, DB-5 Column (60 m)
Abundance (counts x 10⁶)
5.0
4.0
3.5
3.0
2.5
2.0
1.5
1.0
0.5
α-Pinene
Camphene
Δ³-Carene
Limonene
Camphor
trans-β-Caryophyllene
α-Gurjunene
Aromadendrene
Viridiflorene
Palustrol
Ledol
HO
H
O
15
15
20
25
30
35
40
45
50
tR, min

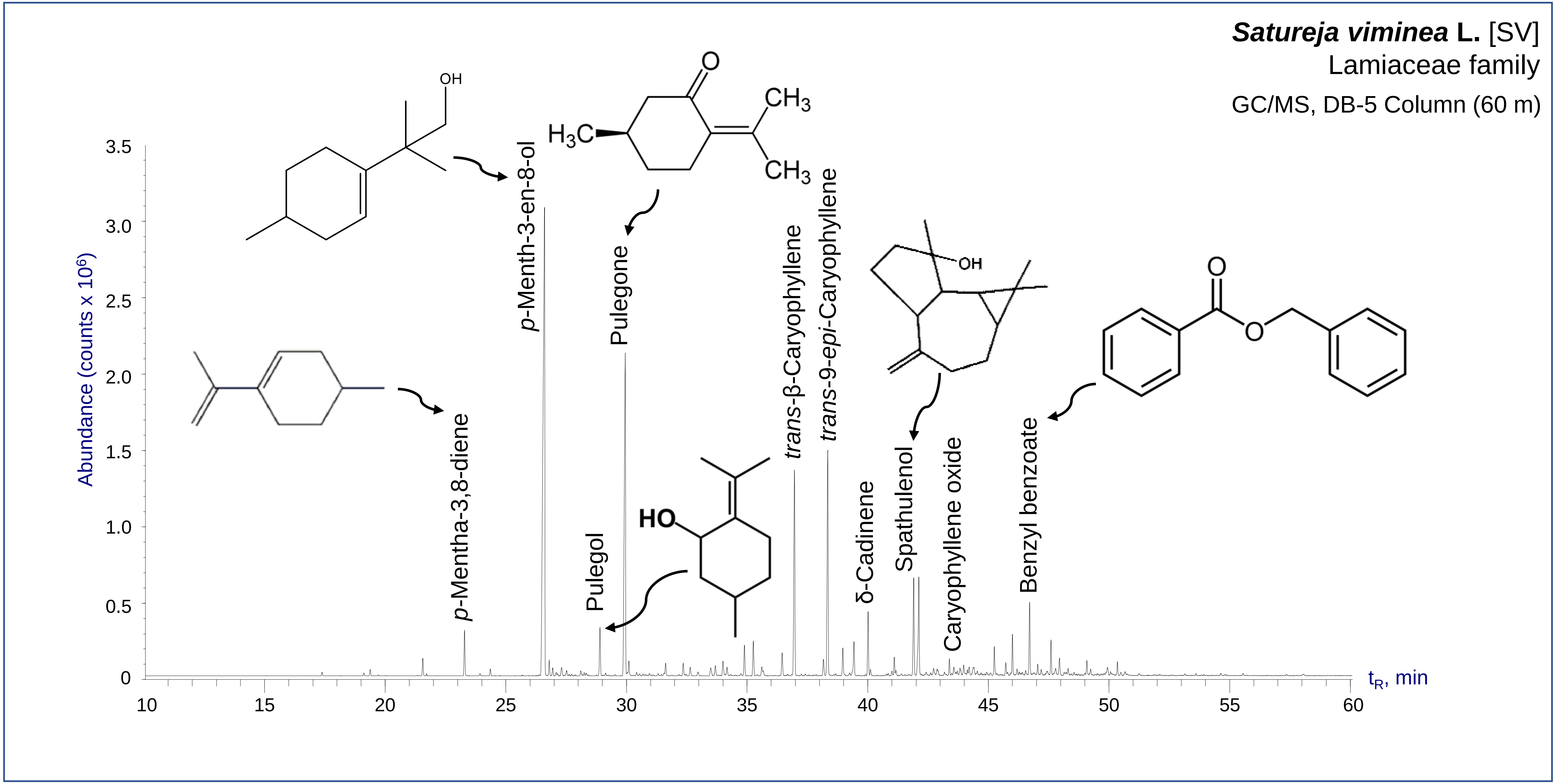

Satureja viminea L. [SV]
Lamiaceae family
GC/MS, DB-5 Column (60 m)
Abundance (counts x 10^6)
3.5
3.0
2.5
2.0
1.5
1.0
0.5
0
10
15
20
25
30
35
40
45
50
55
60
tR, min
p-Mentha-3,8-diene
p-Menth-3-en-8-ol
Pulegol
Pulegone
trans-β-Caryophyllene
trans-9-epi-Caryophyllene
δ-Cadinene
Spathulenol
Caryophyllene oxide
Benzyl benzoate
OH
O
H3C
CH3
CH3
HO
OH
O
O

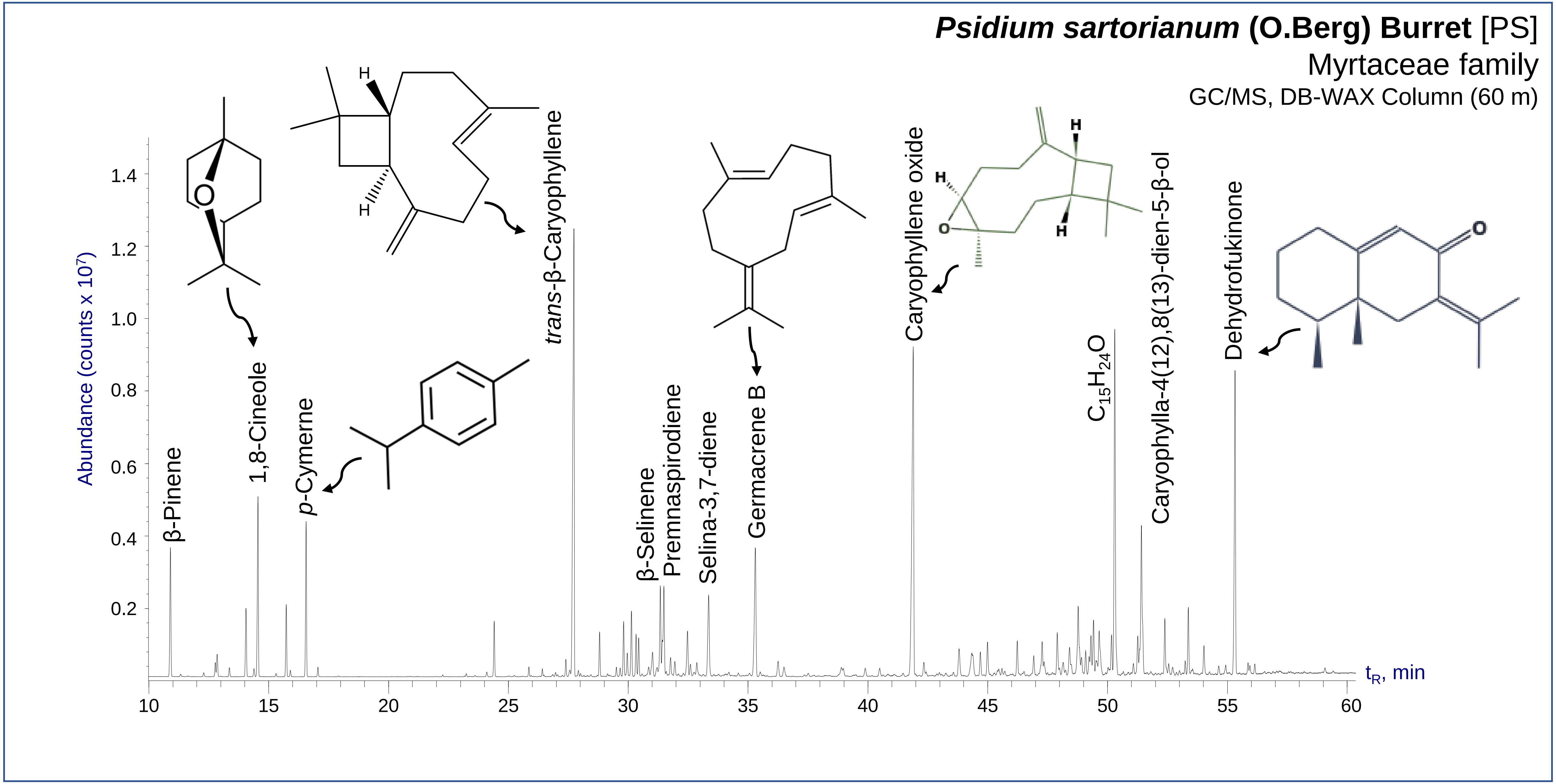

***Psidium sartorianum* (O.Berg) Burret** [PS]
Myrtaceae family
GC/MS, DB-WAX Column (60 m)
Abundance (counts x 10^7)
1.4
1.2
1.0
0.8
0.6
0.4
0.2
β-Pinene
1,8-Cineole
p-Cymene
trans-β-Caryophyllene
β-Selinene
Premnaspirodiene
Selina-3,7-diene
Germacrene B
Caryophyllene oxide
$C_{15}H_{24}O$
Caryophylla-4(12),8(13)-dien-5-β-ol
Dehydrofukinone
H
O
10
15
20
25
30
35
40
45
50
55
60
t_R, min

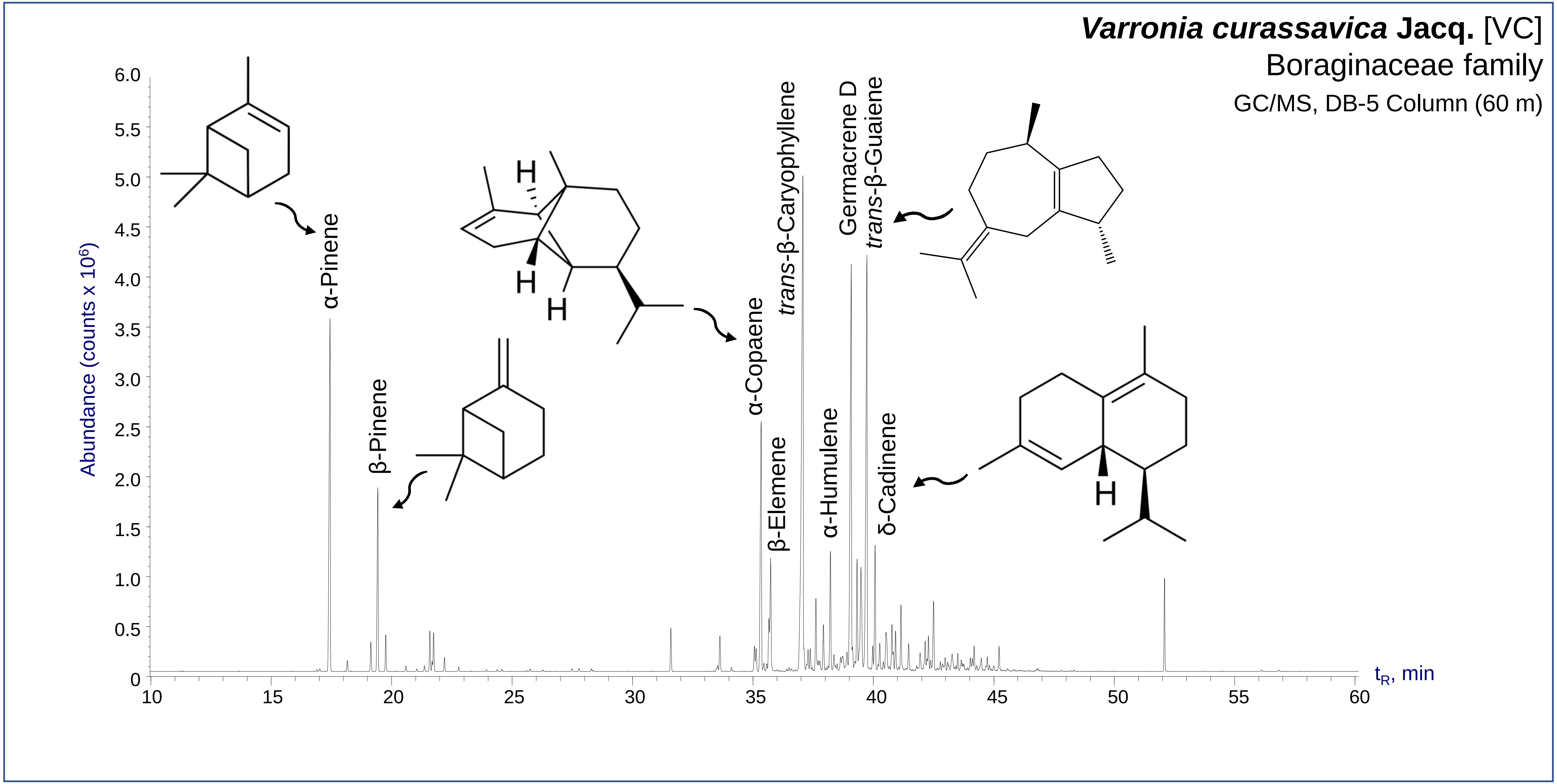

Varronia curassavica Jacq. [VC]
Boraginaceae family
GC/MS, DB-5 Column (60 m)
Abundance (counts x 10^6)
tR, min
α-Pinene
β-Pinene
α-Copaene
β-Elemene
trans-β-Caryophyllene
α-Humulene
Germacrene D
trans-β-Guaiene
δ-Cadinene
H
H
H
H
6.0
5.5
5.0
4.5
4.0
3.5
3.0
2.5
2.0
1.5
1.0
0.5
0
10
15
20
25
30
35
40
45
50
55
60

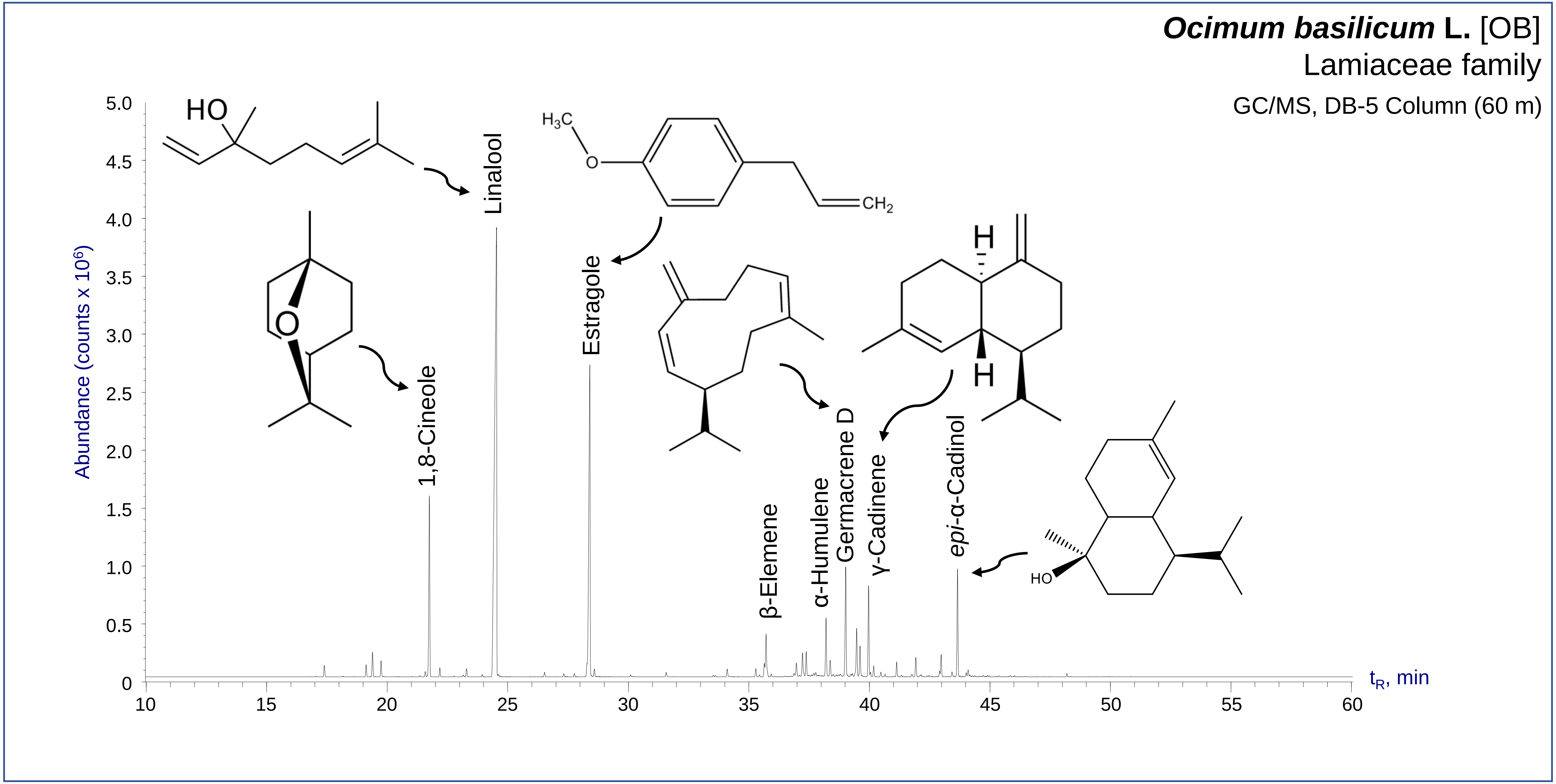
Ocimum basilicum L. [OB]
Lamiaceae family
GC/MS, DB-5 Column (60 m)
Abundance (counts x 10^6)
5.0
4.5
4.0
3.5
3.0
2.5
2.0
1.5
1.0
0.5
0
10
15
20
25
30
35
40
45
50
55
60
tR, min
HO
H3C
O
CH2
H
H
HO
O
1,8-Cineole
Linalool
Estragole
β-Elemene
α-Humulene
Germacrene D
γ-Cadinene
epi-α-Cadinol

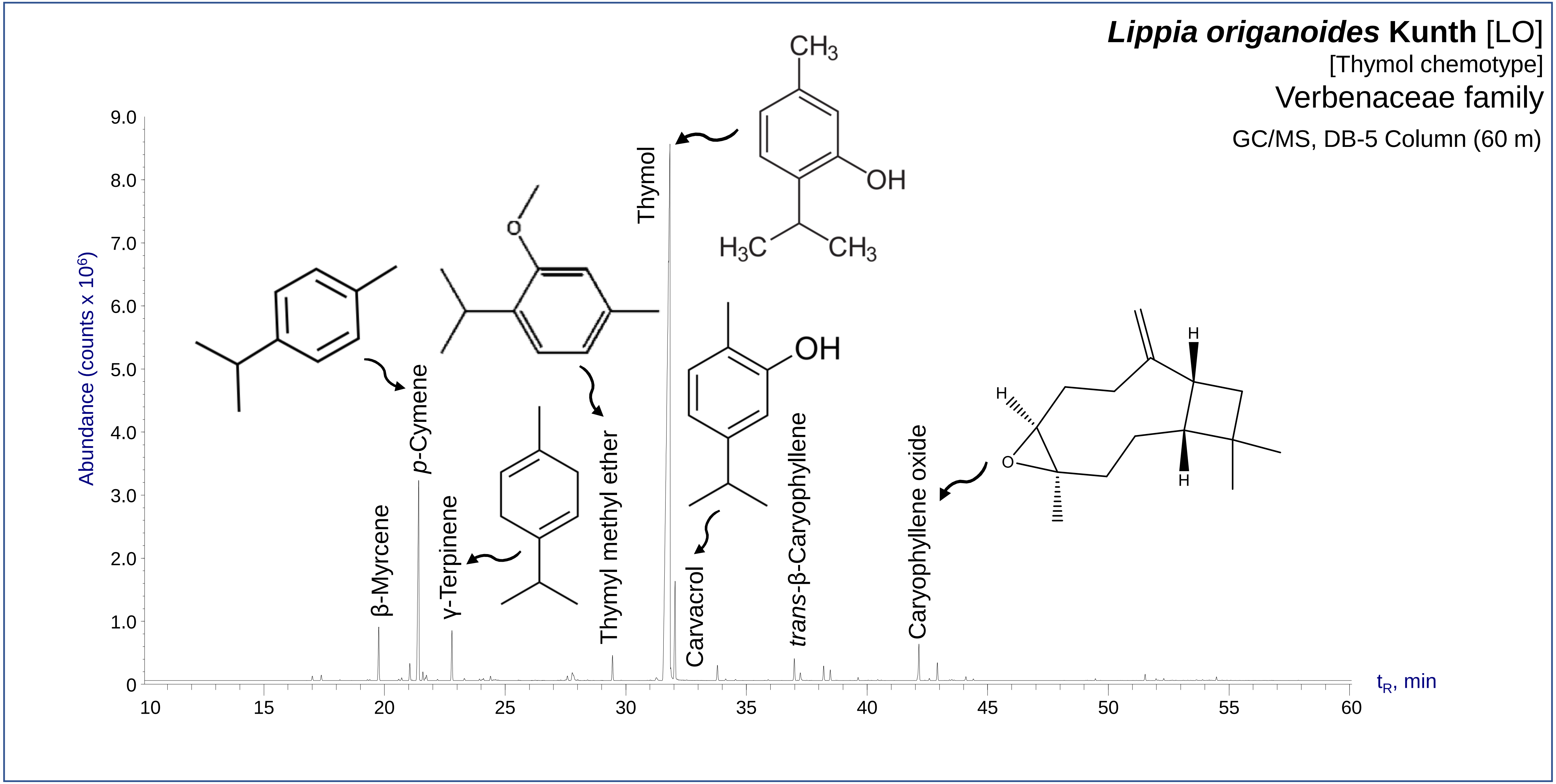
Lippia origanoides Kunth [LO]
[Thymol chemotype]
Verbenaceae family
GC/MS, DB-5 Column (60 m)
Abundance (counts x 10^6)
tR, min
β-Myrcene
p-Cymene
γ-Terpinene
Thymyl methyl ether
Thymol
Carvacrol
trans-β-Caryophyllene
Caryophyllene oxide
CH3
OH
H3C
O
H

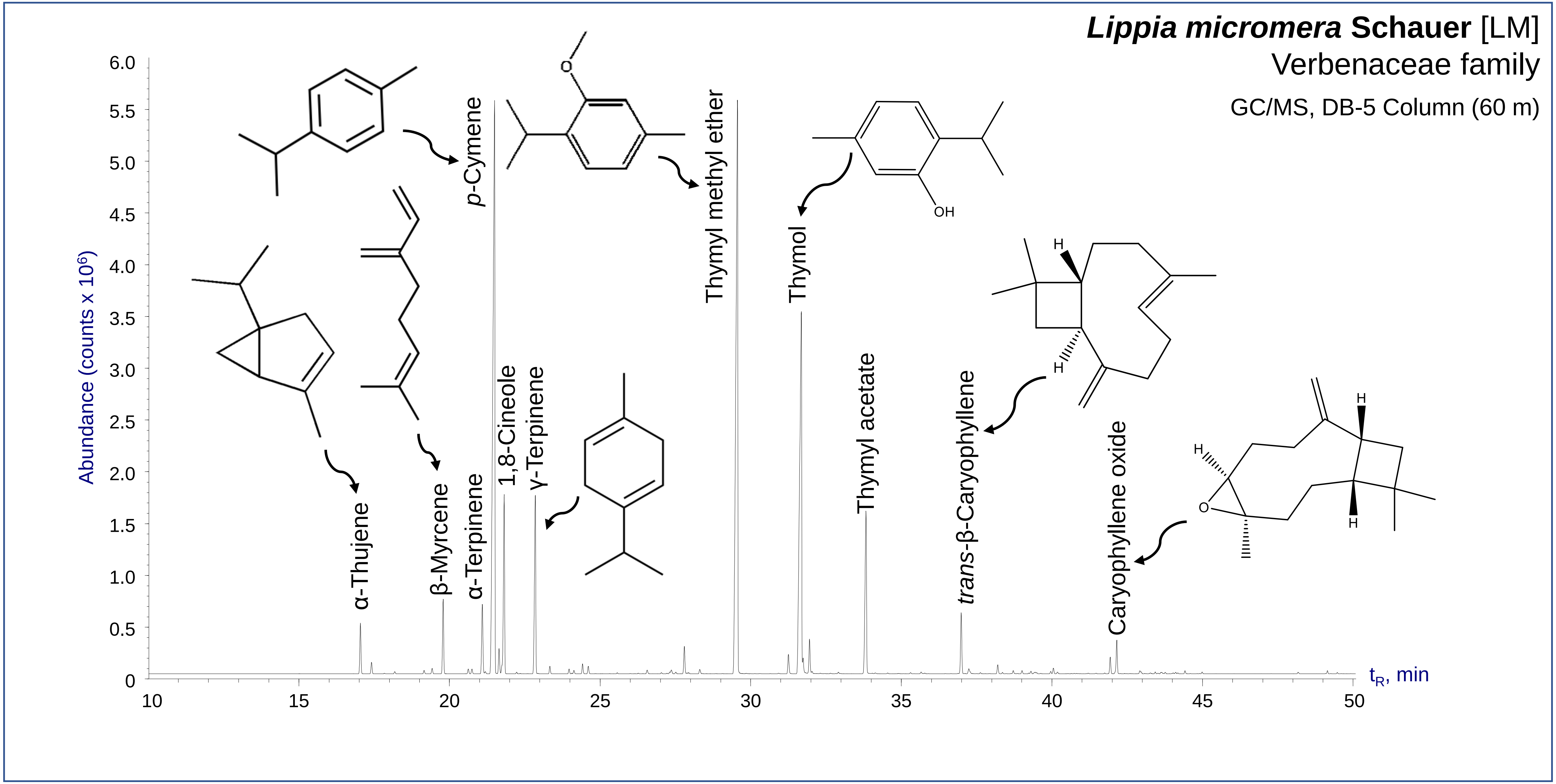

Lippia micromera **Schauer** [LM]
Verbenaceae family
GC/MS, DB-5 Column (60 m)
Abundance (counts x 10^6)
t_R, min
α-Thujene
β-Myrcene
α-Terpinene
p-Cymene
1,8-Cineole
γ-Terpinene
Thymyl methyl ether
Thymol
Thymyl acetate
trans-β-Caryophyllene
Caryophyllene oxide
OH
O
H

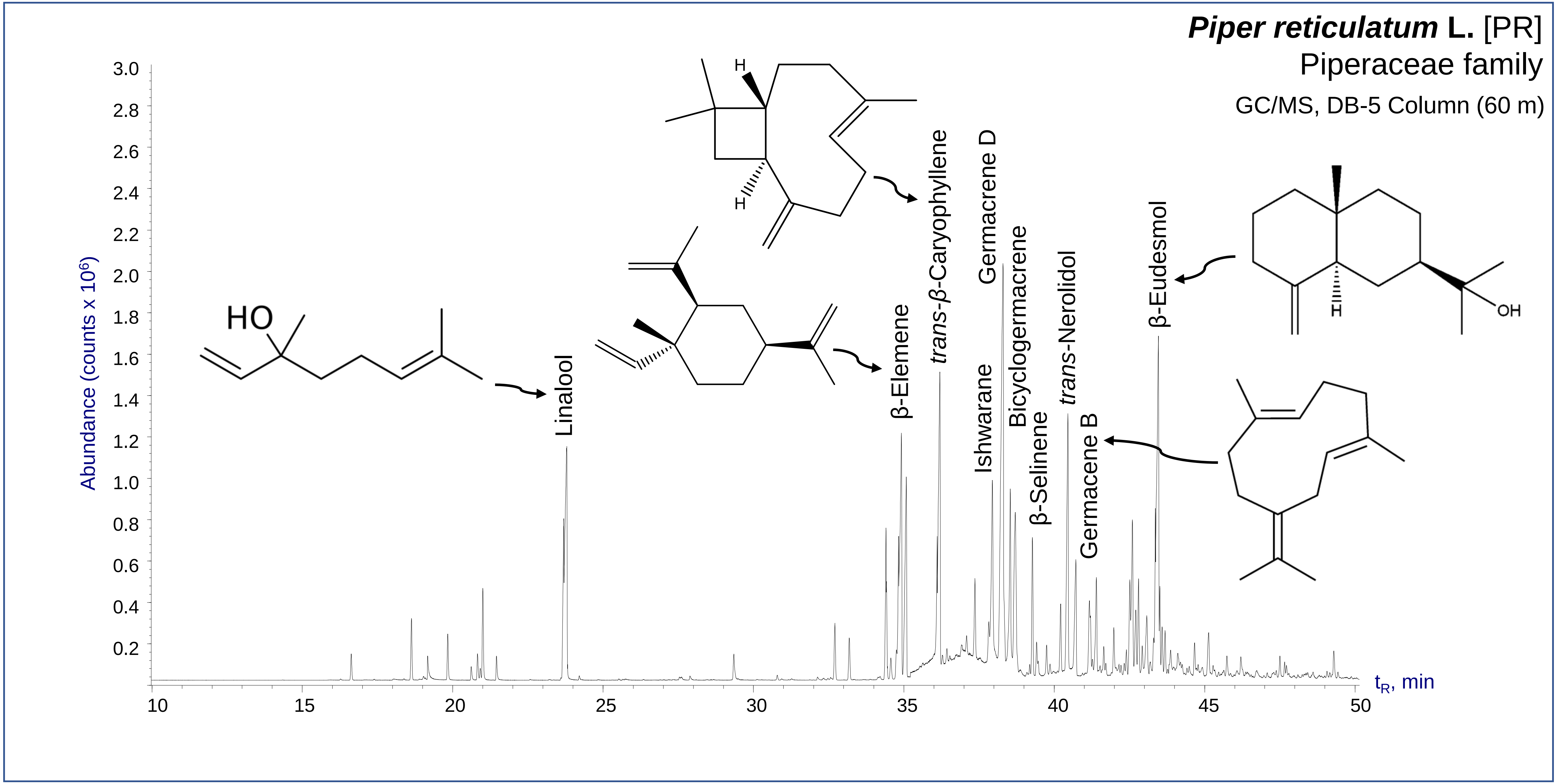

Piper reticulatum L. [PR]
Piperaceae family
GC/MS, DB-5 Column (60 m)
Abundance (counts x 10^6)
3.0
2.8
2.6
2.4
2.2
2.0
1.8
1.6
1.4
1.2
1.0
0.8
0.6
0.4
0.2
10
15
20
25
30
35
40
45
50
tR, min
HO
H
H
H
OH
Linalool
β-Elemene
trans-β-Caryophyllene
Ishwarane
Germacrene D
Bicyclogermacrene
β-Selinene
trans-Nerolidol
Germacrene B
β-Eudesmol

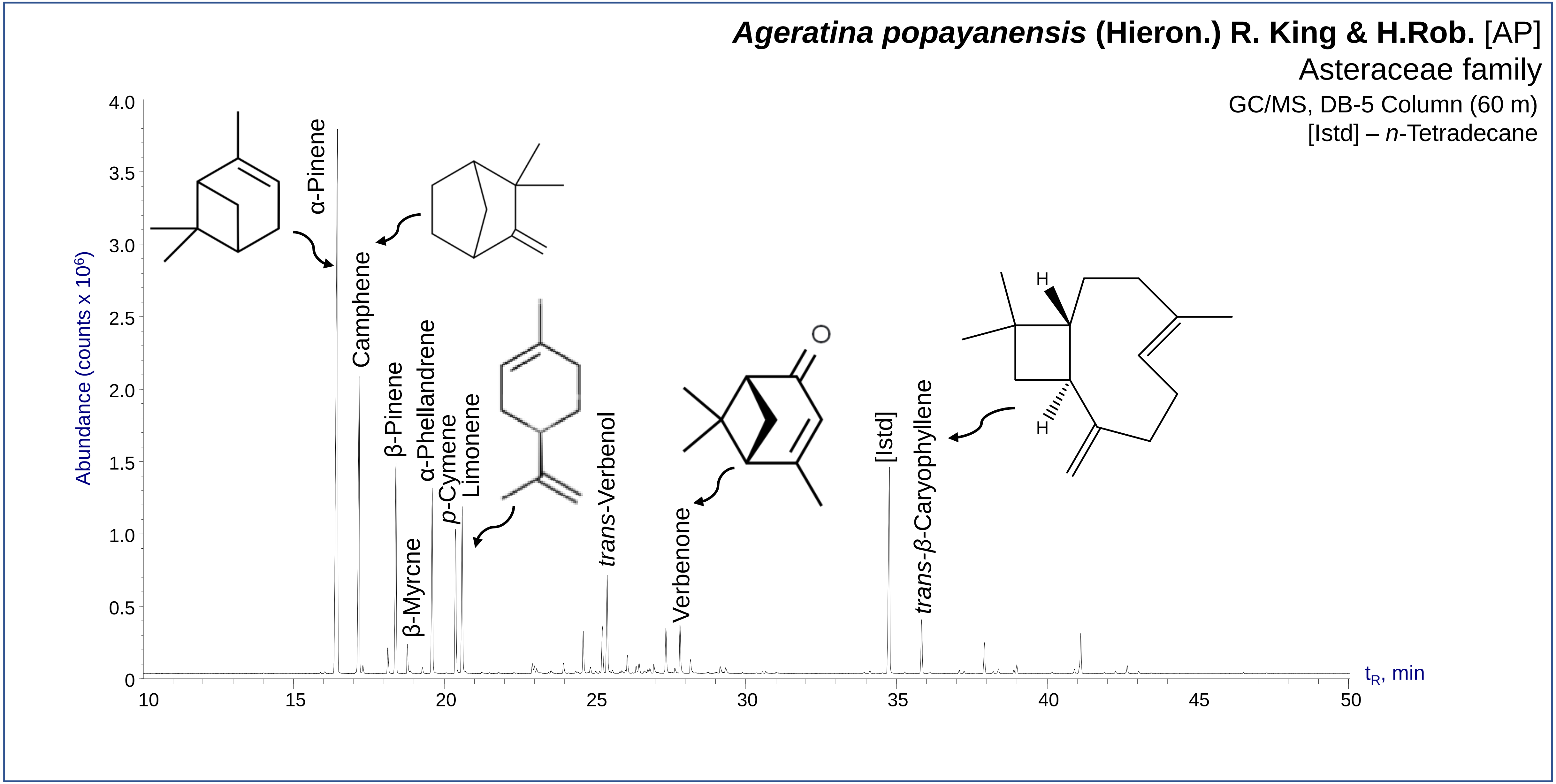

Ageratina popayanensis (Hieron.) R. King & H.Rob. [AP]
Asteraceae family
GC/MS, DB-5 Column (60 m)
[Istd] – n-Tetradecane
Abundance (counts x 10^6)
4.0
3.5
3.0
2.5
2.0
1.5
1.0
0.5
0
α-Pinene
Camphene
β-Pinene
β-Myrcne
α-Phellandrene
p-Cymene
Limonene
trans-Verbenol
Verbenone
[Istd]
trans-β-Caryophyllene
H
H
O
t_R, min
10
15
20
25
30
35
40
45
50

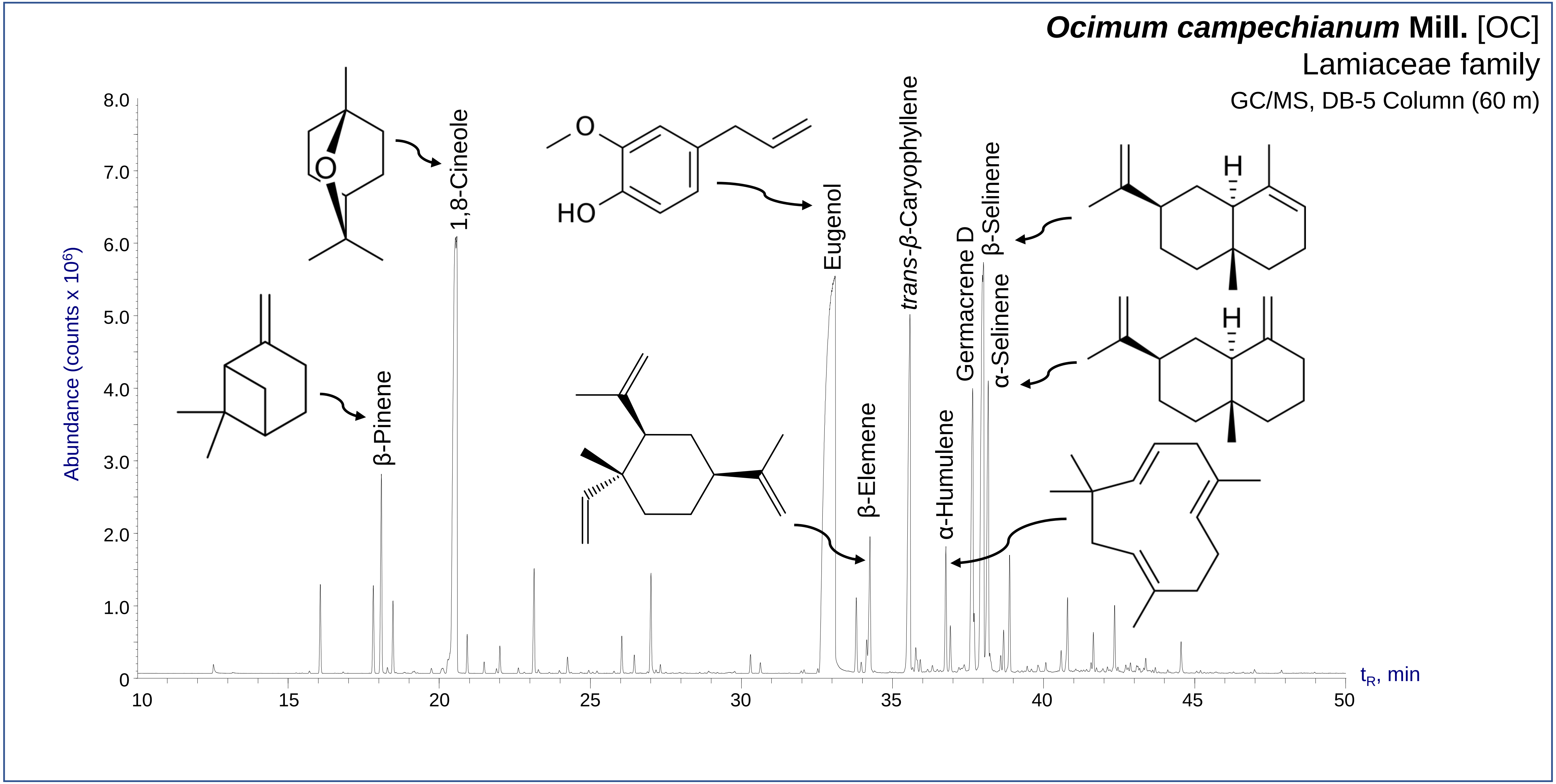

Ocimum campechianum **Mill.** [OC]
Lamiaceae family
GC/MS, DB-5 Column (60 m)
Abundance (counts x 10^6)
t_R, min
8.0
7.0
6.0
5.0
4.0
3.0
2.0
1.0
0
10
15
20
25
30
35
40
45
50
β-Pinene
1,8-Cineole
Eugenol
β-Elemene
trans-*β*-Caryophyllene
α-Humulene
Germacrene D
β-Selinene
α-Selinene
O
HO
H
